# An application of activity theory to the “problem of e-books”

**DOI:** 10.1016/j.heliyon.2020.e04982

**Published:** 2020-09-19

**Authors:** Karen Kirby, Muhammad N. Anwar

**Affiliations:** Department of Computer and Information Sciences, Northumbria University, UK

**Keywords:** Education, Information science, Activity theory, E-books, E-readers, Academic learning, Metacognition, Autoethnography, Reading

## Abstract

The “problem of e-books” is defined as the difficulty improving the adoption rates of e-books by students. The adoption rates of e-books for academic use remain low, and research into the reasons for this have resulted in inconclusive findings. Factors such as student perception, and variations in experimental methodology and technology, contribute to difficulties in generalising findings and establishing conclusive causes for this problem. To better understand the causal factors for low adoption rates and the student's experience with ereaders and digital text, an investigation was conducted by the lead researcher as a student enrolled in a postgraduate course. The experiment was designed using e-book and digital text documents on an ereader for academic study and the results analysed with the framework of Activity Theory. This methodology allowed exploration of the problem within the authentic experience of a student to examine the effects of this social environment on ereader and e-book use.

Analysis of the work domain was conducted and a comparative assessment of the observed effect of using the digital documents on an ereader compared with the paper book. Findings show that attempts to apply self-regulation and metacognitive learning techniques within the activity using the ereader were abandoned due to breakdowns in operations, and that this resulted in a perceived lower quality of achievement. The effect on the processes used by the student were extreme and were observed to be highly dependent on the student's use of specific learning strategies. The experimental methodology employed in this investigation enabled identification of the role of the social environment in the use of course documents on an ereader for academic study. The functionality of the ereader was such an extremely poor fit with the observed academic processes that a redesign approach for ereader and e-book technology is proposed as a solution to the low adoption rates of e-books.

## 1 Introduction

E-books sales represent 30% of the fiction publishing market, 22% of the academic publishing market and 95% of academic journal purchases (Publishers Association, 2018). Even though there are clear differences between the academic and leisure market sectors, the same e-book and ereader products are offered for each sector. After the initial hype over the introduction of the ereader, both the ereader and e-books met with resistance to adoption and sales in recent years are slightly declining (Publishers Association, 2018).

The “problem of e-books” can be described as three separate issues that form the basis of research studies into academic use of e-books.

1. Why are the adoption rates for student use of e-books and ereaders so low?
2. What are the effects of using digital text or ereaders for learning and academic study and how does this relate to adoption rates?
3. What changes can be made to e-book and digital text delivery to improve the adoption rates?

Studies into the “problem of e-books” have resulted in inconclusive and conflicting findings in regards to the reason for low adoption rates and Wilkinson (2015) states that “researchers are interpreting the issue of what constitutes the “e-book problem” differently”. The effects of digital text and ereaders on comprehension (Halamish and Elbaz, 2019; Lenhard et al., 2017; Sidi et al., 2017; Stole and Anne Mangen, 2020), the benefit of changes to teaching methodology and library delivery associated with e-book use have been widely explored as pathways to improving adoption rates (Casselden and Pears, 2019; Raynard, 2017; Sage et al., 2019; Yuan et al., 2017). The disparity in results is contributed to by a mismatch between student perception and observed behaviour (Baron et al., 2017; Kurata et al., 2016; Liverpool University, 2010; Sage et al., 2019), and variation in technology and study methodology that makes comparison and relation of results problematic (Delgado et al., 2018; JISC, 2017; Williams and Showers, 2014). Wilkin and Underwood (2015) conducted an examination of studies on the use of e-books in academic libraries and concluded that the problem could be classified as “wicked” (Churchman, 1967), due to the difficulty defining the problem space, the complex social issues and the inability to generalise the results. Within Churchman's framework (1967) he notes that a “wicked” problem often requires a different approach for investigation as it may be resistant to diagnosis by standard methods of analysis.

To explore the effect of using an ereader for academic study, an experiment was designed and conducted as a comparative analysis of the effect on academic study of the use of an ereader and a paper book. Autoethnography (Anderson, 2006) and Activity Theory (Vygotsky, 1978) were applied in a novel methodological application with supporting concepts from information theory (Belkin, 1980). Testing was conducted using both an ereader and a paper book within the context of postgraduate course requirements for two concurrent course modules. Variables of technological variation, user perception, test environment and student learning strategies were kept constant, providing internal validity for the causal property of the ereader. In this way, it was possible to isolate and examine the effects caused by the ereader. The complexity of the work practices employed by the student and the detailed impact of the change to digital text was examined and linked to the variation in findings on students' use of e-books and their low adoption rates.

### 1.1 Literature studies

Scientific studies focusing on the factors underlying academic resistance to ereaders and e-books are sparse (deNoyelles et al., 2015; Wilkinson, 2015) and widely varied in technique and orientation (Delgado et al., 2018; Singer and Alexander, 2017). In a meta-analysis of 54 research published between 2000-1017, Delgado et al. (2018) note that within findings of existing literature “media effects are inconsistent”. For example, while studies have found that students don't mind the loss of formatting and colour (Richardson and Mahmood, 2012), other investigations indicate that the lack of formatting in ereaders is a significant problem (Brook et al., 2010) and background research supports the use of formatting in navigating documents for learning as very important (Lovelace and Southall, 1983; Mangen et al., 2013; Rothkopf, 1971). Variation in comprehension when using ereaders and digital text provides some of the most inconclusive findings among studies, particularly in relation to the perception of comprehension. Students frequently report that they perceive that comprehension is lower when using ereaders (Baron et al., 2017; Weisberg, 2011), however in Sage et al. (2019), Halamish and Elbaz (2019), and Nie et al. (2011) the students surveyed reported that the “experience from an ereader is similar to reading from a book”. When investigating comprehension from electronic text, Singer and Alexander (2017), Rockinson-Szapkiw et al. (2013) and Gregory (2008) found that students perceived that their comprehension improved when reading from e-books. Studies have shown internal and external disparity of student perceptions of e-books, ereaders and related experiences (Baron et al., 2017; Gregory, 2008; Sage et al., 2019) and when comparing qualitative and quantitative results Liverpool University study (2010) found that “perceptions about e-book usage, particularly among students may not be accurate”.

Wilkin and Underwood (2015) observe that the variations in study methodology and technology makes it “impossible to arrive at a reliable set of generalisable conclusions” about the factors behind the adoption rates of ereaders and Singer and Alexander (2017) found on review of existing research that a lack of definitions created difficulty in comparing studies. Recent studies of the effect of digital text by Baron et al. (2017), Delgado et al. (2018) and Sidi et al. (2017) have shown a significant effect on student behaviour of variations in time constraints, and this indicates the importance of the incorporation of the academic social environment in research design. The methodology of this investigation was designed to address two particular factors affecting literature findings: student perception and understanding the effect on study processes of the use of e-books within the social environment.

#### 1.1.1 Background theory

A brief overview of theoretical concepts utilised in the analysis of experimental results is presented here.

##### 1.1.1.1 Information behaviour

Information Behaviour describes human behaviour used in seeking and utilising all forms of communication in a wide range of settings and scenarios (Wilson, 2000). For this research, information use behaviour is relevant as the interpretation and use of written information for learning as well as information seeking behaviour in working with the ereader and paper book. Belkin (1980) used information behaviour to develop the concept of a knowledge gap which he defines as an “Anomalous State of Knowledge” or ASK. According to Belkin, when using information for learning, the student will recognise a difference between the knowledge required for the established purpose and the knowledge currently held. The reader/learner will then seek to resolve this ASK using the written information and will utilise information use and seeking processes such as scanning, reading, recognition of key works, evaluation and decision making. When learning from a paper book, these steps are facilitated by spatial navigating using the formatting of the reading material (Lovelace and Southall, 1983; Mangen et al., 2013; Rothkopf, 1971) and the effect of the change in media on these aspects of information behaviour will be explored in this research.

Kuhlthau (1993) uses information seeking behaviour to describe the steps taken in the case that a learner abandons resolution of an ASK. In this instance Kuhlthau noted that the subject had emotional responses to searching for the required information and that, in the absence of confidence that this information need can be resolved, the learner abandons the search. Kuhlthau noted that this occurs when a number of attempts to resolve the information need, including by restructuring the search, do not return fruitful results and the user concludes that they will not be able to resolve the ASK. This experimental environment includes the pressures of the academic social setting to incorporate the authentic parameters required to generate this behaviour. Information theory (Belkin, 1980; Kuhlthau, 2019; Wilson, 2000) will be used to recognise the exercise of this behaviour as well as the setting of goals in relation to knowledge gaps and information selection and retrieval using the different media.

##### 1.1.1.2 Reading

Foertsch (1998) describes reading as follows: “The process involves intellectual and complex tasks that may encompass the use of several cognitive strategies for achieving specific objectives”. In a student study by ChanLin (2013) titled “Reading strategy and the need of e-book features”, interviews were used to structure hypotheses that were then tested through think-aloud protocols, surveys and statistical analysis. The findings confirmed that students read in different patterns to a leisure reader when using e-books, and study participants demonstrated pre-reading text subconsciously and focusing on selections of interest when performing academic reading. Academic reading varies significantly in goal and implementation from leisure reading and Gier et al. (2011) found that college students use “multiple reading strategies” to meet their “numerous” reading requirements.

##### 1.1.1.3 Learning

Separate from comprehension, the concept of learning is very relevant for investigations into the adoption of e-books for academic use. Where it has been possible to assess comprehension in laboratory tests, learning is a more complicated application of skills and techniques that students employ in response to course requirements and social ambitions. Learning strategies are described by Gurung et al. (2010), as including “time management, goal setting, selecting what to study, how, and where; taking good notes; reading and self-testing”. The positive correlation of the application of meta-cognitive techniques such as self-regulation with academic performance has been established by a number of studies (Hattie, 2009; Garner, 1987; Paris et al., 1991) and the research of Brown et al. (1981) included the importance of “checking, planning, asking questions, self-testing and monitoring”.

Intrusions into workflow caused by breakdowns have the potential impact of requiring goal-oriented action through executive function and it is noted by Souza et al. (2018) that attention required for demanding tasks impairs working memory. The impairment as a function of time affects the retention interval in a ratio known as “cognitive load”.

#### 1.1.2 Activity theory

The model of Activity Theory is powerful as an enormously useful lens for diagnosis, particularly for complicated human systems where motivation, creativity and interactions with an artefact or tool are core factors. The framework of Activity Theory (AT) offers advantages in addressing the “problem with ereaders”: in establishing a focus on the goal for the activity system processes, the actions are viewed in their relevance to achieving the goal and the effect of the varied tool on these actions can be observed. The goal and outcome are determined by the subject within the social context and this experiment was conducted with assessable academic requirements. Engestrom (1999) describes the importance of the object within Activity Theory as “the object of an activity is it's true motive”. Using this description, the nature of the activity is defined by the content of the object. In the model described by Leont'ev (1978), the object exists in 2 forms, the independent existence and the subject's mental image of the object so that “man's activity is regulated by mental images of the reality” (Engestrom, 1999). Leont'ev (1981) also described the role of the social-historical context in generating and motivating the activity and the outcome requirements are balanced by the perceived system conditions, the constraints of the rules and the influence of the community. As Leont'ev (1978) notes: “motives, goals and conditions of the activity must be perceived, understood, retained and reproduced by his memory”. As the activity progresses, there is a constant intricate adjustment of this perception and weighing of an acceptable outcome with consideration of the community and rules.

**Figure 1:**
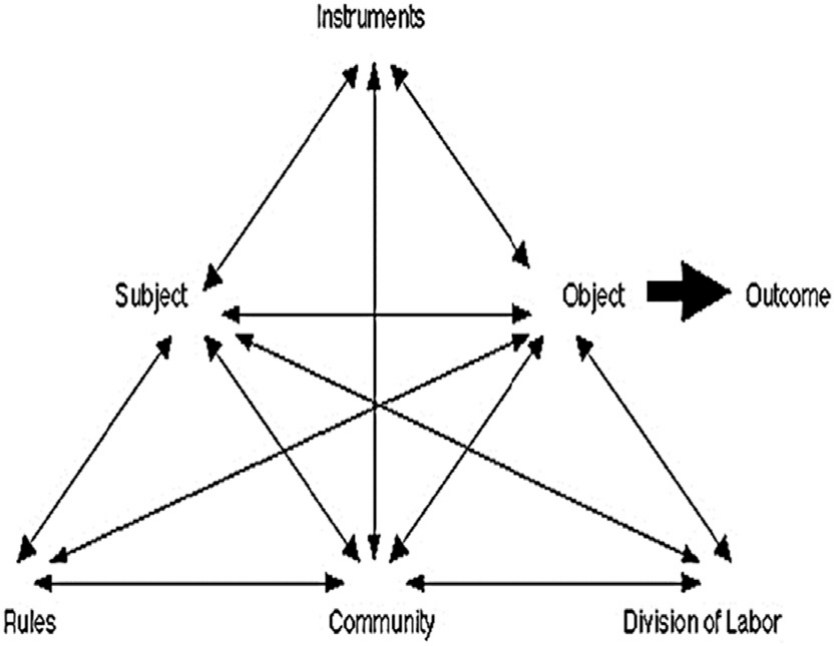
2nd Generation Activity Theory (Engestrom, 1999). Adapted under CC-by-SA-3.0, Bury (2012).

The processes occurring within the activity system are described as actions and operations (Leont'ev, 1978). Actions are conscious processes that are created and enacted by the subject in order to achieve a goal and these translate to Engelbart's (1963) executive function when considering cognitive load and working memory. The actions are constructed of a number of operations that can be subconscious to the subject (Leont'ev, 1978), which reduce the cognitive load of the activity. The second generation of the activity theory model was constructed by Engestrom (1999) and includes the impacts of the community, the division of labour and any rules the system may need to adhere to as shown in Figure 1.

The understanding of the conditions, including the functionality of the tool, are critical to successful transformation of the object to the ideal goal state (Leontyev, 1981).

##### 1.1.2.1 Activity theory and Human Computer Interaction

Activity Theory has been utilised in application for studying Human Computer Interaction (HCI), user design and Computer Supported Cooperative work since the 1990's (Kaptelinin and Nardi, 2009). Nardi, Kuutti and Kaptelinin in particular, have explored it's use as a framework that considers the role of information technology as the mediating tool, and the psychological aspects of user engagement (Nardi, 1996). Since this early work, the goal orientation and incorporated social environment of Activity Theory has been utilised in HCI studies to diagnose interaction and adoption issues related to the implementation of new technology in a wide range of social environments (Al-Huneini et al., 2020; Blin and Munro, 2008; Lu et al., 2018; Sun, 2016). Activity theory analysis in HCI has no formalised practice methodology and for these studies a wide range of supporting theories and processes were employed to inform relationship analyses and observe interactions.

Bedny and Meister (1997) proposed modern applications of activity theory including studies of situational awareness (Bedny and Meister, 1999) and user design. Within this research, Bedny (2004) found that that “..any activity has a recursive, loop structure, organised according to the principles of self-regulation in which feedback mechanisms that evaluate performance are decisive”. Understanding and being able to utilise the system processes is critical for self-regulation and Bedny noted the importance of the system conditions and planning for goal-directed activity. Tikhomirov (1999) and Babaeva et al. (2013) also described the importance of goal-setting in “self-regulation of thinking and the formation of cognitive structures” within activity systems. In the creative activity of learning, students respond to information needs, use strategies and balance priorities.

In giving context to Information Technology problems (Kuutti, 1999), analysis using Activity Theory can be used to explore the cognitive processes of decision making and prioritisation in relation to goal achievement. This is of benefit when the social environment is suspected as a critical factor and in a study of academic behaviour among doctoral candidates (Lei and Hu, 2019), activity theory analysis was used to understand the students' decision making when interacting with their environment to solve academic problems. In a review of 109 HCI investigations, Clemmensen et al. (2016) found that researchers frequently developed theory for use in Activity Theory analysis. The work of Davydov (1999), Tikhomirov (1999) and Bodker (1996) were utilised in this research for their applicability to the issues and methodology of this investigation, and are discussed below.

###### 1.1.2.1.1 Davydov

In an analysis of the interdisciplinary use of Activity Theory, Davydov (1999) recognised continuing problems in determining a methodological approach and proposed 4 stages of study applications. These stages have been interpreted and utilised for analysis of the results of this research. Davydov considered that: “The goals of activity manifest themselves as images of the foreseen result of the creative effort” and described the transformation of the object to the ideal state in the following steps:

• Identification of the object content.
• Defining the content structure and their transformations and interrelations, methods of exchange.
• Defining the ideal goal.
• Significant features of the activity.

Further confirming the importance of the subject's perspective of the system conditions and self-regulation, Davydov (1999) stated that for the purposes of the transformation, the subject seeks to “understand the conditions of origination of integral systems”.

###### 1.1.2.1.2 Tikhomirov

In his development of activity theory, Tikhomirov (1999) noted the relevance of the activity theory model to creative activity and described additional objects in the form of the new product of the activity and the image of the future object. He proposed that creativity is the development of possibilities in the form of these new images. Within this research, the image of the future object was the ideal knowledge state that the subject worked towards and the new product is the transformed knowledge state that is evaluated against the goal and iteratively updated. Goal setting can be voluntary or involuntary and is a result of evaluating incoming information and progress, including the “nonachievement of the anticipated results” (Tikhomirov, 1999). The application of the model of activity theory to creative activities shows a complex iteration and generation of actions, modification of goals and assessment of priorities in the transformation of the object to the ideal state.

###### 1.1.2.1.3 Bodker

Bodker (1996) used breakdowns and focus shifts developed by Winograd and Flores (1986) and Activity Theory to analyse interaction with information systems in a project with the Danish Labour Service (DLS). To better understand the processes and problems of the employees using a new computer system, interviews, videotapes of use, ethnography and interaction records were compiled and inspected for breakdowns and focus shifts. The project sought to identify the real problems encountered when using the technology on the job and to link the processes employed by staff to the particular motives of the subjects. Bodker defined breakdowns within the activity system as disrupted work, and focus shifts as a change in focus causing a deliberate action (Bodker, 1996). Using these definitions, Bodker investigated the activity to determine “whether the breakdowns are causing shifts in focus, and whether these breakdowns are the result of design problems with the tool”. Bodker stated that for an activity system to function effectively the tools as mediators should not be “themselves objects of our activity use” and this understanding recognised the importance of the subject's ability to utilise the tool using subconscious operations. When evaluating the design of a mediating tool it follows that the “artefact works well in our activity if it allows us to focus our attention on the real object and badly if it does not” (Bodker, 1996). These definitions and interpretations have been incorporated for assessment of the ereader within this experiment.

#### 1.1.3 Analytic autoethnography

Examining the experience of the subject in situ is always challenging and ethnography is often employed to address information gathering requirements in the real environment (Preece et al., 2015). Hughes et al. (1995) states that as an experimental method “ethnographic accounts present the user behaviour as it actually exists, rather than idealized pictures of how the user “ought” to approach a task”.

Autoethnography is “the ultimate participant in a dual participant-observer role” (Merton, 1988) and enables focus on identifying the factors relevant to the problem space through the informed position of the researcher (Cunningham and Jones, 2005; Reed-Danahay, 1997). In an investigation into the stigma experienced in the social environment of competitive swimming in Australia, McMahon et al. (2019) utilised analytic autoethnography to facilitate access to the social environment as lead researcher/subject. This methodology enabled observation and recording of a socially generated effect by the informed subject with an added “vantage point” (Anderson, 2006). For this investigation, analytic autoethnography is used with activity theory to explore the use of an ereader within an actual academic social context. Academic study is a predominantly cognitive activity, and to understand the effect of the tool on learning strategies and decision making, access to the internal processes of the student was facilitated by autoethnography.

##### 1.1.3.1 Generalising using activity theory

Analytic autoethnography differs from ethnography in the use of modelling and analysis to generalise the results beyond the experience of the researcher and is a distinct “sub-genre” of analytic ethnography (Anderson, 2006). Autoethnography is affected by the bias implicit in the user as the observer and subject “bear the signature and voice of personal interpretations” (Duncan, 2004). To address this issue within the experiment, the interactions and decision-making processes were journalised and coded using information theory concepts (Belkin, 1980; Kuhlthau, 2019; Wilson, 2000) and modelling with activity theory enables some generalisation of results and external validity beyond the single sample set (Anderson, 2006).

## 2 Materials and methods

### 2.1 Research methodology

The experimental design addresses the issues identified from literature of student perception, technological variation, and the social environment to identify the effect of using e-books.

1. Testing was observed in a real academic environment using AE and subsequent analysis using the AT framework incorporated the effects of the social environment on processes, goal-orientation and decision making (Leont'ev, 1978). The experiment was conducted by the lead researcher as an enrolled postgraduate student and testing was completed within course timeframes. This design allows observation of the issues of time constraints and the relative importance of work raised by Delgado et al. (2018), Sidi et al. (2017) and other studies (Lenhard et al., 2017; Nichols, 2018; Stole and Anne Mangen, 2020). Sidi et al. (2017) and Delgado et al. (2018) found in separate studies that the effect of an imposed time limit produced a reduction in comprehension that was more significant when reading from electronic text. In the study by Sidi et al. (2017), the effect of the time constraint was found to vary, depending on the student's perception of the importance of the task and Kerr and Symons (2006) found that additional time was required when using e-books, affecting efficiency. Investigation as a participant researcher subject to academic pressures enabled exploration of this line of enquiry.
2. The differences in study and reading patterns between individual students are significant (Brown et al., 1981; Gier et al., 2011). When multiple students are used for testing and the results compared within a sample set, the variation in patterns of use presents a significant variable affecting observation of the effect of the ereader. For example, while some students may report that the annotation available for e-books is inadequate (Baron et al., 2017; Nie et al., 2011), other students will not utilise this process in the same way and will not report any deficiencies when using it (Richardson and Mahmood, 2012). This generates complexity in the assessment of the effect of using digital text on annotation, and other individualised learning practices. Using the same student for a comparative study of the effect of the two types of media, removes the impact on results of varied student practices. To confirm the relevance of the example, the experimental results are placed within the set of literature findings.
3. The experiment is confined to academic use only. In addition to evaluating the use case within the academic social environment, this design aspect acknowledges the studies by ChanLin (2013), Brook et al. (2010), Delgado et al. (2018) and Sidi et al. (2017) in which behavioural patterns used when reading for leisure and academic study are found to vary significantly. The experiment examines e-book use, and the use of journal and smaller digital text documents separately, in response to significant differences in sales statistics (Publishers Association, 2018). Qualitative research findings also indicate variations in use between e-books and shorter documents (Hobbs and Klare, 2015; Swain, 2010; Tracey, 2018; Zhang et al., 2017).
4. As Delgado et al. (2018), Liverpool University (2010) and Gregory (2008) found, students' perceptions and bias in regards to ereaders and e-books has contributed to inconsistent findings, and student responses to usage of the product has produced survey results that are not supported by observed reading behaviour (Halamish and Elbaz, 2019; Kurata et al., 2016; Sage et al., 2019). The experiment is designed to produce differential results that are observed by comparing the use of the two media types within constant experimental parameters. Observational bias is minimised through normalisation of this effect in the comparative experiment.
5. The effect of the ereader was isolated by maintaining consistency in experimental parameters other than the mediating tool. Activity Theory was used to identify the causal relationship between the effect of the ereader and the work domain addressing the recommendations of Delgado et al. (2018), Singer and Alexander (2017), Baron et al. (2017), and Sidi et al. (2017). Autoethnography provided detail into the cognitive decision making and metacognitive learning processes which was mapped to goal-directed actions and workflows that could be directly compared.

### 2.2 Product review

The Kobo Aura One (Kobo, 2020) was selected as it provided advanced functionality and enabled importing of the most document types of any ereader on the market. While the Kindle ereader was more popular, this was a due to the larger market share for the Kindle store which would have no bearing on the experiment.

A hardware ereader was selected to take advantage of screen technologically specifically designed for extended reading and the smaller, lighter dimensions (Table 1). The large range of imported document types available on the Kobo ereader provided the best possible outcome for Section 2 of the experiment which tested multiple document formats. The Kobo Aura One offered the most functionality within the Kobo range other than the newer Kobo Clara (Kobo, 2019), which was found to be too small to accommodate sufficient text at an average font size.

**Table 1:** Comparison of ereaders.

	Kobo Aura One	Kindle Oasis	Kindle app	iBook
Screen technology	E-Ink Carta/ComfortLight Pro	E-Ink Carta	LCD^1^	
ppi	300	300	326	
Dimensions	1404 × 1872	1264 × 1680	1536 × 2048	
Size (inches)	7.8	7	8	
Weight	230 g	194 g	300 g	
Import document types	Epub, epub3, pdf, mobi, txt, html, rtf, cbz, cbr, jpeg, gif, tiff, bmp	Azw, epub, pdf, mobi	Azw,epub, mobi, pdf	Epub, pdf
Choice of font	Yes	Yes	Yes	Yes
Popularity	2	1	-	-
Annotation	For epub	For Azw	For Azw	Epub,pdf
Release Date	Sep 2016	June 2015	March 2009	April 2010

1 The dimensions of an iPad mini (5^th^ gen) tablet were used for both applications.

### 2.3 Structure

The experiment commenced in 2018 and was completed in 6 months over 25 study sessions. The enrolled courses were distance postgraduate subjects of a technical nature and the utilised set textbooks were “Interaction Design” (Preece et al., 2015) and *Statistics and Business Intelligence* and the e-book “Big Data, Big Analytics” (Minelli et al., 2013).

The experiment was divided into the following sections:

1. *Comparative assessment of the use of a paper book and e-book on the Kobo ereader for an assigned course text.*

Chapter one of the set textbooks for two separate modules were read to completion in a number of study sessions. Journal notes were made during or immediately subsequent to reading and were typed onto a laptop, phone or tablet as available depending on location. Occasionally notes were handwritten and transcribed.

2. *Use of the Kobo ereader for assigned course materials including lectures.*

To consider the use of the Kobo ereader for the entire requirements for one course module, the Kobo was used for all lectures and course provided documents for the semester. This section of the experiment explored the use of smaller documents with varied formatting.

3. *Analysis of results was conducted using Activity Theory framework.*

Davydov's (1999) four stages of investigation were interpreted to analyse and model the activity from the experimental results.

• **Stage 1** - identify the object content.
• **Stage 2** - detailing the object structure/condition. What are the processes/actions that change it? How are transformations affected in the environment? The autoethnography compiled during the experiment provided this information and breakdowns and focus shifts were observed.
• **Stage 3** – identifying and examining the image of the ideal object. Comparison with the existing object, detailing the processes employed to try and achieve the ideal object.
• **Stage 4** – identify significant features of the Activity System.

## 3 Analysis

### 3.1 Comparison study

#### 3.1.1 Stage 1 - Identifying the activity

The activity is identified by the content of the object (Davydov, 1999; Leont'ev, 1978) and, for the student, the object undergoing transformation is the *knowledge state* of the subject. On considering the annotation and note-taking that is incorporated when using the book or ereader, the knowledge state must include written work. The object is thus defined as the internal (conceptual), and external (written) knowledge state of the user. This description then leads to identification of the activity as *academic work*. Interactions with the object are mediated using the reading material on an ereader or paper book.

**Figure 2:**
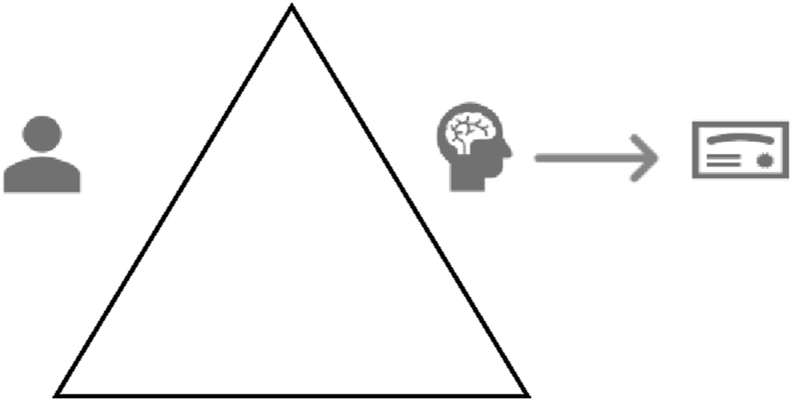
Academic work Activity.

**Figure 3:**
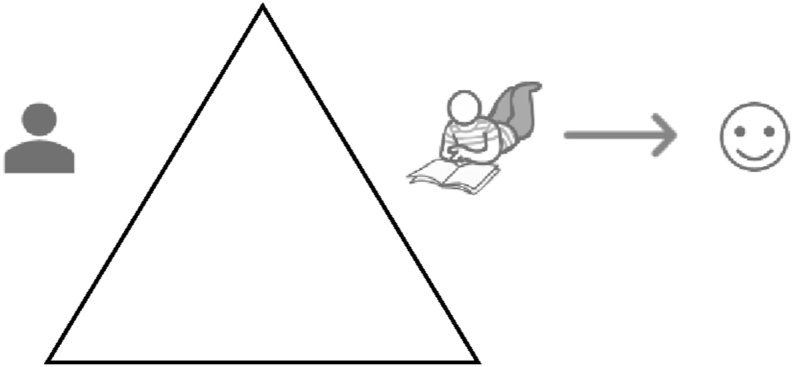
Leisure reading activity.

Identification and inspection of the goal and outcome of this activity (stage 3), revealed that the outcome desired was a positive academic result. This included good grades, esteem from colleagues, and self-regard motivated by personal ambitions (Figure 2). This identification clearly contrasted with the use of an ereader or paper book for leisure reading. In the latter instance, the object of the activity is a leisure experience. The subject is using a fiction book to mediate a window of leisure time to create a good reading experience (Figure 3). The desired outcome for this activity is a positive leisure experience and the activity is described as *reading for leisure*. It is clear on initial consideration of the content of the object, that leisure reading, and academic work are separate activities.

#### 3.1.2 Stage 2 - Detailing the object structure/system conditions

With the activity now defined, the structure of the knowledge state, the processes of transformation and the conditions of the system are investigated. The existing knowledge state was the understanding of the concepts required for the outcome, as well as any existing written work. While it was clear that transformation of the knowledge state is achieved by reading and note-taking, it was unknown to what extend other actions and operations would facilitate the transformation. This detail was provided by the autoethnography. The range of actions and operations, as well as the conditions and utilisation of the tools were revealed through the journalised investigation.

##### 3.1.2.1 Identifying operations and conditions

Table 2 lists the operations that were utilised within the activity and whether these were adequately supported by the media tools. These assessments were made by reviewing the autoethnography and identifying each distinct process that was attempted. Where the operation was completed, support was considered satisfactory. A determination of unsatisfactory was made where further attempts at using the process were abandoned due to difficulty or unavailability. For “Highlight text”, sufficient functionality was present that this operation was able to be completed, though without the flexibility of writing in a paper book.

**Table 2:** Identifying operations.

Supported Operations		
Operations	Kobo	Paper
Read text	Yes	Yes
Scan Page	No^1^	Yes
Change page	No	Yes
Scroll/turn page	No	Yes
Use index	No	Yes
Underline text Highlight text	No Yes	Yes Yes
Write in book	No	Yes

1 Operations are listed as “No” if significant breakdowns were experienced when trying to use this them.

By not adequately supporting a number of operations conducted during academic work, all actions other than reading were problematic for academic work using the Kobo. Breakdowns and focus shifts were experienced at multiple points during operations using the Kobo. The failing operations then moved from the subconscious to executive action (Bodker, 1996; Engelbart, 1963), incurring greater cognitive load and disrupting the thought trail (Souza et al., 2018). Information searching behaviours of scanning, contextualising, identifying keywords and spatial orientation were frequently disrupted or could not be completed. In one example, when attempting to locate content read during a previous session, scrolling was attempted using the ereader for searching via keywords and scanning of the text. The scrolling took a long time and the search was abandoned when the material was not quickly located.

#### 3.1.3 Stage 3 – The ideal object

##### 3.1.3.1 Identifying the ideal object

When considering the ideal object, the community and rules influencing the activity were identified, as well as the student's understanding of the conditions under which the activity would be conducted. In this activity, the ideal object was the Ideal Knowledge State (IKS_u_), and this was established as the image of the desired future knowledge state object (Tikhomirov, 1999). IKS_u_ was the student's perception of the Knowledge State required to achieve the desired outcome (Outcome_i_) (1.1).

(1.1) IKS_u_ → Outcome_i_

The student then identified a knowledge gap in the comparison with the student's initial Knowledge State, KS_0_ and IKS_u_^1^ (1.2).

(1.2) IKS_u_ – KS_0_ = knowledge gap

This knowledge gap is consistent with Belkin's (1980) Anomalous State of Knowledge (ASK). The student then translated this ASK to an information need, and initiated information seeking behaviour using the available written material. To assess the utility of the written material in addressing the knowledge gap (ASK) the student conducted an assessment of the book. This corresponded to the subject assessing the conditions of the activity.

(1.3) CK_0_ → CK_1_

The assessment of the written material updates the initial Content Knowledge (CK_0_)of the book to a new state of Content Knowledge (CK_1_)about the book (1.3).

For this experiment, this assessment was conducted in the first study session. The evaluation of the Content Knowledge included a range of questions such as: *What is the content of the book like? How many pages? How in depth is the information? How does it relate to the rest of the book and the course? How engaging is the material?* To address these queries in relation to the Content Knowledge of the written material, information seeking behaviour was employed and information retrieval actions included scanning, keyword search, review of depth and volume of the content were initiated using the book and ereader in separate sessions.

In the interaction with the paper book, the information need for the Content Knowledge was met. For example, the index was easily found and scanned to determine the number of chapters and the number of pages in each chapter. The formatting was observed through flipping pages in the chapter, and the relevance of the chapter to be read in relation to the other chapters in the book was established using chapter headings in the index. The depth of the content was reviewed in the text using section headings and scanning, and this was related to the desired Ideal Knowledge State (IKS_u_). The difficulty of the reading was determined by quickly reading some sentences from within the chapter. Use of the paper book was successful in providing information to update CK_0_→CK_1,_ and this updated Content Knowledge (CK_1_) was used to determined that the paper book was likely to provide an acceptable IKS_1p_ and thus Outcome_1p_ (1.4).

(1.4) $$\begin{array}{ccccc} {\text{CK}}_{\text{0}} & \to & {\text{IKS}}_{\text{u}} & \to & {\text{Outcome}}_{\text{i}}\\ ↓ & & ↓ & & \\ {\text{CK}}_{1} & \to & {\text{IKS}}_{1\text{p}} & \to & {\text{Outcome}}_{1\text{p}} \end{array}$$

For the Kobo, this result was very different. In this instance, the information need in relation to the Content Knowledge was not met due to unsuccessful actions caused by disrupted operations. On trying to review the index, seven pages needed to be scrolled, which was prohibitive to effectively searching. The easily scanned information per page was limited in quantity and minimally formatted, with no colour and little differentiation. It was difficult to retain details in short-term memory while scanning to enable assessment of the relevance of chapters to each other, and scrolling was too slow for easy refreshing of memory. Scrolling pages to view the chapter content was also too slow to allow easy assessment, and a scan of the nature of reading could not be put into a broader context within the chapter or book. Difficulty obtaining this information led to abandoning the information retrieval goal in relation to the Content Knowledge. This demonstrated the behaviour described by Kuhlthau (1993) as a number of different attempts were made that were unsuccessful, and this led to a determination that the retrieval exercise would not be successful, and the search was abandoned. This effect is interesting as the time constraints for the information retrieval and the desire for the Ideal Knowledge State were established by the participation in an assessable course. Consideration of these requirements and abandonment of this process can be clearly attributed to the use of the ereader as a tool in this instance. CK_0_ was not updated, and knowledge of the book remained a simple understanding of the type of text. Without an update to CK, the student was left with a state of uncertainty about the conditions of the activity (2.1).

(2.1) $$\begin{array}{ccccc} {\text{CK}}_{\text{0}} & \to & {\text{IKS}}_{\text{u}} & \to & {\text{Outcome}}_{\text{i}}\\ ↓ & & ↓ & & \\ {\text{CK}}_{\text{0}} & \to & {\text{IKS}}_{1\text{e}} & \to & {\text{Outcome}}_{1\text{e}} \end{array}$$

The difficulty, length and relevance of the textbook content are key aspects of the tool and form part of the conditions of the system (Brown et al., 1981; Engestrom, 1999). As noted by Engestrom (1999), a good understanding of the conditions of the system is a core requirement for the subjects' ability to achieve the desired outcome. As CK_1_ cannot be identified, the perception is that:

(2.2) $$\begin{array}{ccc} {\text{IKS}}_{1\text{e}} & \text{<} & {\text{IKS}}_{1\text{p}}\\ {\text{Outcome}}_{1\text{e}} & \text{<} & {\text{Outcome}}_{1\text{p}} \end{array}$$

The expectation of the student was that use of the ereader tool will yield a lower academic result than that for the paper book (2.2).

Engestrom (1999) described the constant weighing of acceptable outcome that is required for successful learning and this was observed in the decision making, and subsequent abandonment, that occurred when reading with the ereader. This analysis using Activity theory reveals the importance of the perceived conditions and outcome when attempting to use an ereader for academic work in the perceived futility of efforts to resolve problems within timeframes, and the resultant perceived lower outcome.

##### 3.1.3.2 Progress

As Tikhomirov (1999) notes, assessment of progress towards the ideal object is critical. Without this understanding, self-regulation is not possible and this is essential for achieving the ideal goal (Bedny, 2004) and also for learning (Hattie, 2009; Garner, 1987; Paris et al., 1991). In the case of the paper book, a review was conducted on the commencement of each study session to determine retention of the previous reading (3.1). It is detailed as follows:

(3.1) $$\begin{array}{ccc} {\text{KS}}_{\text{0}} & & \text{CK}1\\ ↓\text{C} & & ↓\\ {\text{KS}}_{1} & ↔ & {\text{CK}}_{2} \end{array}$$

In the prior session, KS_0_ was updated with Content - C - to the new KS_1_ and the Content Knowledge of the book (CK_1_) was updated during this process to CK_2_. On commencing the next session, KS_1_ is evaluated against CK_2_^2^ by scanning the read pages and testing the content of the updated Knowledge State (KS_1_). Using the paper book, an example of this assessment consisted of flipping the previously read (five-ten) pages, noting the headings and considering whether these were now known concepts, and also reading some parts of the text and testing KS_1_ for familiarity. Page flipping was heavily utilised and the coloured, formatted panels that delineated the content were used for identifying topics and the relationship of content. This review was conducted to ensure that the planned content (C) was fully or sufficiently incorporated into KS_1_ before continuing. In addition to testing the knowledge state for progress updates at the commencement of reading sessions, reviews of concepts, or points of reference, were conducted during reading sessions. With the paper book this consisted of locating the needed information by flipping pages, noting page numbers against the approximate time during the reading session that the content was encountered, and scanning the text to refresh the concept or re-reading sections of it fully.

When reading with the Kobo, neither the commencement review of the progressed KS_1,_ nor the in-session testing of incorporated reading was possible due to difficulty in scanning and scrolling the e-book. To confirm the update to the Knowledge State from the prior reading session, attempts were made to scroll back through the read pages, but this was so slow that the working memory query failed, and the retrieval was abandoned. For attempts to confirm incorporated learning during sessions, less scrolling was required as the information was on immediately preceding pages, but the lack of distinctive formatting and colour, combined with the slow scrolling, meant that few retrieval queries were satisfied. Updates to the KS could frequently not be confirmed and the overall result was that the updates to IKS for the Kobo were almost impossible to confirm or assess.

##### 3.1.3.3 Mediating the object with the tool

For most reading sessions using the paper book, frequent assessments were made to determine the relevance of the book content to the ideal knowledge state. For example, in the second reading session, the content was examined for relevance at the commencement and throughout the session in addition to the previously noted reviews for progress (4.1). The evaluation is represented as follows:^3^

(4.1) $$\begin{array}{ccc} {\text{IKS}}_{1}\;-\;{\text{KS}}_{1} & = & \text{knowledge gap}\\ & & ↕\\ & & {\text{CK}}_{2} \end{array}$$

The updated Content Knowledge (CK_2_) was used to determine how to resolve the knowledge gap through planning and strategy. As Tikhomirov (1999) noted, creative intellectual activity utilises non creative functions of “comparison, reproduction, assimilation and copying” with creative actions of “construction, generation and creation”. All of these actions were utilised here with assessment of the knowledge gap and creation of new actions to resolve it, utilising CK_2_. This process continued iteratively with transformations to KS and CK as new information needs were identified with subsequent knowledge gaps (4.2).

(4.2) $$\begin{array}{ccccccc} {\text{KS}}_{\text{0}} & - & {\text{IKS}}_{1\text{p}} & = & \text{knowledge gap} & ↔ & {\text{CK}}_{2}\\ ↓\text{C} & & ↓ & & & & \\ {\text{KS}}_{\text{x}} & - & {\text{IKS}}_{1\text{p}} & = & \text{knowledge gap} & ↔ & {\text{CK}}_{\text{x}}\\ ↓\text{C} & & ↓ & & & & \\ {\text{KS}}_{\text{k}} & & {\text{IKS}}_{1\text{p}} & = & \text{knowledge gap} & \left(\text{acceptable}\right) & \\ & ↓ & & & & & \\ {\text{KS}}_{\text{k}} & & {\text{IKS}}_{1\text{p}} & & & & \end{array}$$

The processes ceased where the utility of the paper book tool was deemed exhausted or competing priorities and time limited further improvements in the KS_x_. The student determined that further reductions in the knowledge gap were not affordable or warranted. This evaluation of obtaining the desired outcome was visible using coded autoethnography results. An example of this was shown in one session where an assessment of the learning was determined to be not strong. A consideration of the desired ideal knowledge state was made, and the index and book content were reviewed using page flipping and scanning to establish the effect of the minimally learnt knowledge content on the Outcome. It was observed that the topic in question would be covered in more detail in a subsequent chapter and that this could be used to prompt a review of the material at a later time if needed. The Knowledge State was considered sufficiently updated and the study session continued. As the paper book was studied, self-regulation and self-assessment were used to establish progress in transforming the object (KS) to the desired Ideal Knowledge State (IKS_1_). The existing Knowledge State was consistently compared to the desired mental image and the Content Knowledge was updated as required.

To note relevant passages and record ideas for later work, annotations were made in the margins of the paper book, or text was highlighted. This updated the written knowledge state. For the purposes of the course requirement, this included underlining lines of text, placing asterisks beside paragraphs or graphics, making written notes in the margins and also writing notes and queries in an accompanying document. These notes served to identify lines of thought and enquiry, supported learned concepts for review, and contributed to the construction of assessable work.

For the Kobo, the Content Knowledge was not progressable and the achieved CK_1_ was minimal. This led to reduced ability to assess the use of the content of the e-book for addressing knowledge gaps (5.1). Progress towards the ideal using the e-book then became:

(5.1) $$\begin{array}{ccccc} {\text{KS}}_{\text{0}} & - & {\text{IKS}}_{1\text{e}} & = & \text{knowledge gap}\\ ↓\text{C} & ↓ & & & \\ {\text{KS}}_{\text{k}} & - & {\text{IKS}}_{1\text{e}} & = & \text{knowledge gap}\;\left(\text{final}\right)\\ & ↓ & & & \\ {\text{KS}}_{\text{k}} & = & {\text{IKS}}_{1\text{e}} & & \end{array}$$

The knowledge gaps remained unresolved and unknown in relation to IKS_1e_. Without being able to assess updates to the Knowledge State, KS_k_ was uncertain, and the relationship to the outcome was unknown. The self-regulation and self-assessment required for success in learning and activity systems was not available (Engestrom, 1999). In one example of this problem, content was read using the Kobo that was evaluated to be of particular interest in relation to the desired course outcome. An information need was generated in response to determine the depth that the topic would be covered within the textbook. Scrolling forward to satisfy the information need, this query was abandoned due to the scrolling pace and lack of formatting for navigation of topics. Returning to reading, a broader context could not be established for the topic, and a shallower understanding was anticipated.

It was possible to highlight text with the Kobo ereader and this functionality was more convenient using the e-book. However, a reduction in the quantity of work was perceived as this function utilised pre-set options and highlighting could only be performed over text. Notes could be made digitally, and this function was also convenient, and the notes were more legible. However, visibility for attached notes was minimal and these could not be easily located or identified for reference or consultation. Making notes was abandoned, and highlighting was adapted to selecting text only, resulting in a perception of a reduced Knowledge State due to a lower quantity of work when compared with using a paper book.

#### 3.1.4 Stage 4 - Significant features

The analysis has shown that using the ereader results in a perceived lower outcome due to the failed information retrieval processes. The effect of the two tools will now be further examined by considering the variation in the workflows of the activity, the processes employed, and the goals and actions in relation to the affected work domain.

##### 3.1.4.1 Grouping

To identify the core processes implemented during the activity, actions were identified and grouped according to common goals. These goals were recognised as reading, learning, planning and writing, and all actions were classified as belonging to at least one of these goal groups.

The grouping (Table 3) was established through identification of the motivation and context of each action, and by coding the autoethnography accordingly. For example, was made by highlighting text for referral to a colleague for clarification on a concept. The topic was determined to be poorly understood, and the action taken to resolve the knowledge gap was generated by the goal of *Learning*. This action engaged similar operations, but was distinct from the *Writing* group, in which actions were conducted for developing assignment work, and to support other action groups. These actions were used iteratively and, in any combination, and sequence to achieve the desired goal. A failure in any of the actions resulted in varied processes and possible lower achievement of the outcome. This identification of actions and common goal groups allowed mapping of the processes of the experiment when using the paper book and the ereader, and the difference caused by the ereader was revealed. Analysis during Stage 2 showed that most operations experienced significant breakdowns and focus shifts when using the Kobo ereader.

**Table 3:** Grouping of actions.

Group	Action	Description	Kobo	Paper
Planning	Information seeking	Review or scan content for relevance to knowledge gap or ability to cover in reading session. Examine next session content.	No	Yes
	Deciding	Weigh reading choice against time and goal.	No	Yes
	Use formatting	To apply focus or decide to stop	No	Yes
	Refer colleague	Select to refer to colleague to advance learning	Yes	Yes
Learning	Focus	Direct attention to specific material	No	Yes
	Refer text	Examine previous information	No	Yes
	Use formatting	identify concepts by formatting	No	Yes
	Test knowledge	Review previous reading	No	Yes
Reading	Scan	Rapid assessment of material	No	Yes
	Read content	Basic forward reading	Yes	Yes
	Orient	Find place using graphics, menus.	No	Yes
	Contextualise	Use book content to contextualise content	No	Yes
	Check progress	Use progress % or review pages	No	Yes
Writing	Highlight text	Use highlighter	Yes	Yes
	Make note	Write in margin	No	Yes

##### 3.1.4.2 Reading

The basic action of “read context” utilised the operation of “read text”, and this action was not disrupted when using either tool. The *Reading* grouping contained a range of related actions that were performed to support “reading” within the academic work activity. It is here that failures were demonstrated, and breakdowns in operations caused significant disruption to most other reading actions when using the Kobo. The majority of failures were experienced when attempting to “orient” or “scan” through text which was impacted by slow scrolling and reduced formatting.

As noted by Foasberg (2011), ereaders are best designed for basic linear reading which is the main requirement of leisure readers, while academic reading consists of complex reading strategies (Gier et al., 2011). The cognitive load on students when reading to learn is very high (Foertsch, 1998; Mangen et al., 2013), creating a significant burden for working memory. Reading strategies can be used to alleviate this (Gier et al., 2011), and the inability to implement the range of actions that supported this goal reduced the capability of the subject to offset this burden.

##### 3.1.4.3 Planning

*Planning* occurred in every study session using the paper book and was employed repeatedly. Actions within the planning group incorporated information seeking behaviour in response to numerous knowledge gaps that facilitated learning and self-regulation. Planning also included frequent decision making in weighing the priorities of the community and the rules. This is the most complex behaviour, and the one most directly linked by the motivation of outcome and the perception of progress within the system. A failure in planning created the first critical decision point when using the ereader.

###### 3.1.4.3.1 First session – Paper book

**Figure 4:**
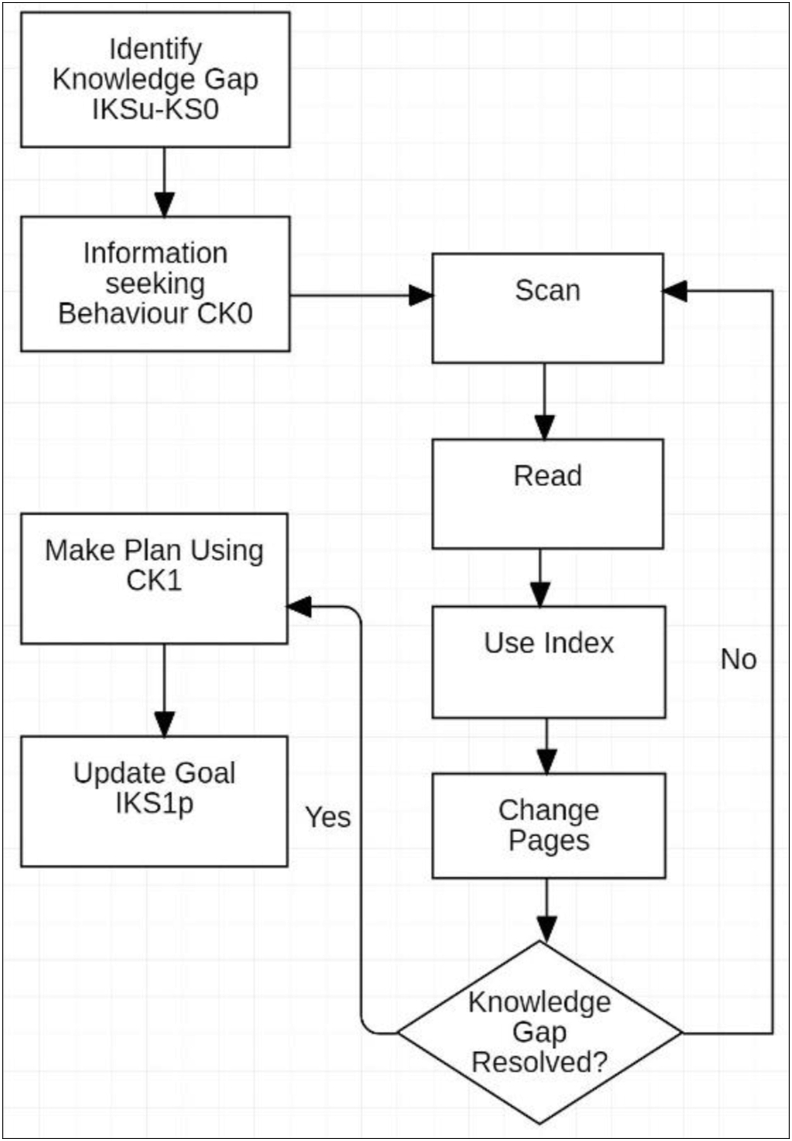
First session paper book.

On commencing reading with the paper book, the first study session was spent planning (Figure 4). The Ideal Knowledge State (IKS_u_) that is perceived to be required for the Outcome_i_ was established as described in Stage 3. The initial Knowledge State (KS_0_) was assessed and a comparison was made between the existing and the Ideal Knowledge State that identified the knowledge gap.

This step utilised information seeking and information retrieval behaviour of scanning and reading, as well as evaluation and decision making. These operations were easily executed with the paper book and the actions within the system were uninterrupted. A study plan for using the book was made using CK_1,_ considering the social context of available time, the difficulty of the material, content and competing priorities. An anticipated reading pace was estimated, with stopping points based on content and format, and an expectation of the possible progress towards IKS_1_ was developed. In this instance, self-regulation was established using a good understanding of the conditions of the activity system and the ability to obtain the IKS_1_ using the paper book. Informed decisions were made in relation to achieving the goal.

###### 3.1.4.3.2 Kobo

**Figure 5:**
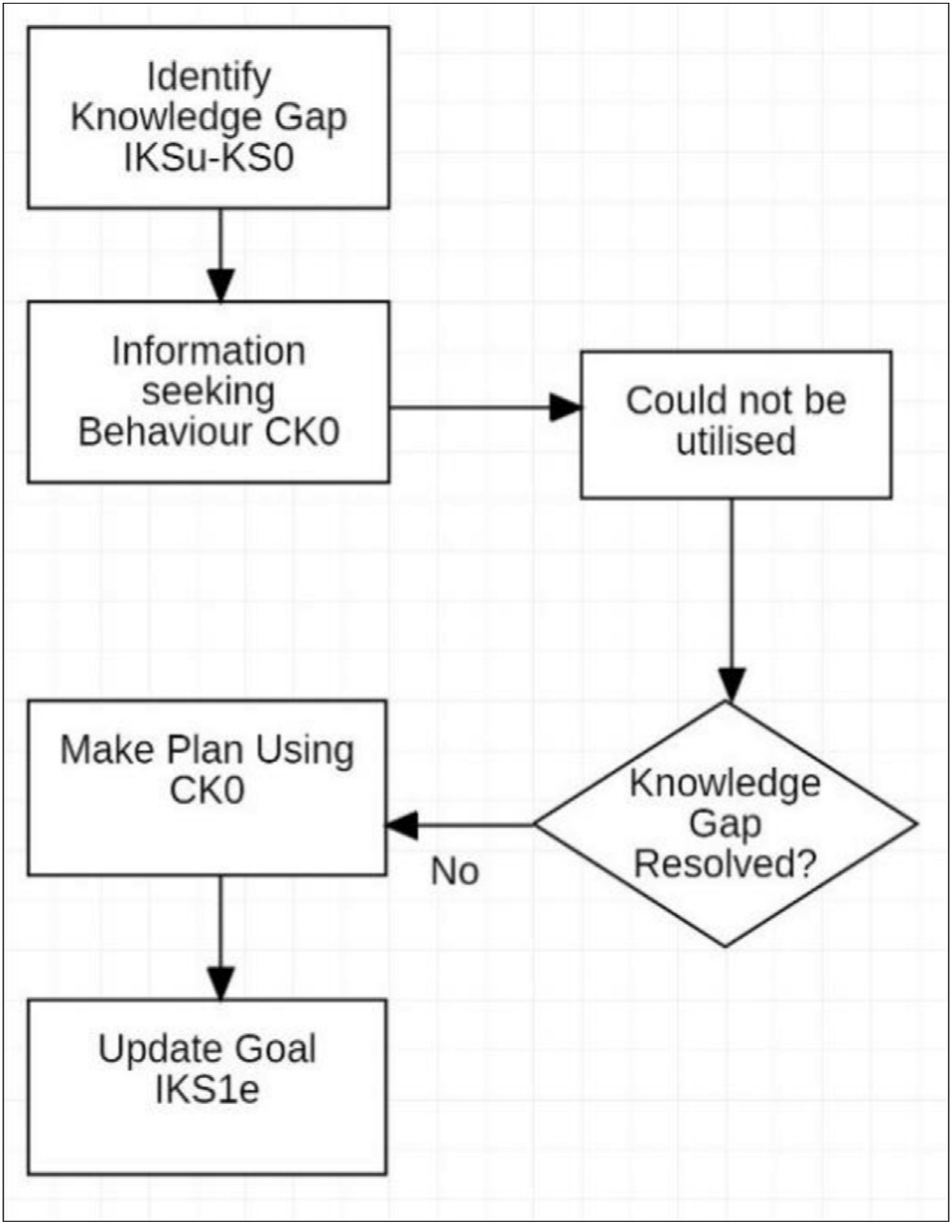
First Session Kobo ereader.

The first study session using the Kobo was significantly abbreviated and analysis revealed a simplified workflow (Figure 5). With difficulty scanning content, consulting the index and changing pages, the information need remained unresolved, which significantly limited planning and self-regulation. It was at this first session that the decision to abandon the tool was likely, and it is proposed that this may correlate to many of the findings of negative student perceptions in previous studies that relate to low adoption rates.

###### 3.1.4.3.3 Subsequent sessions – paper book

**Figure 6:**
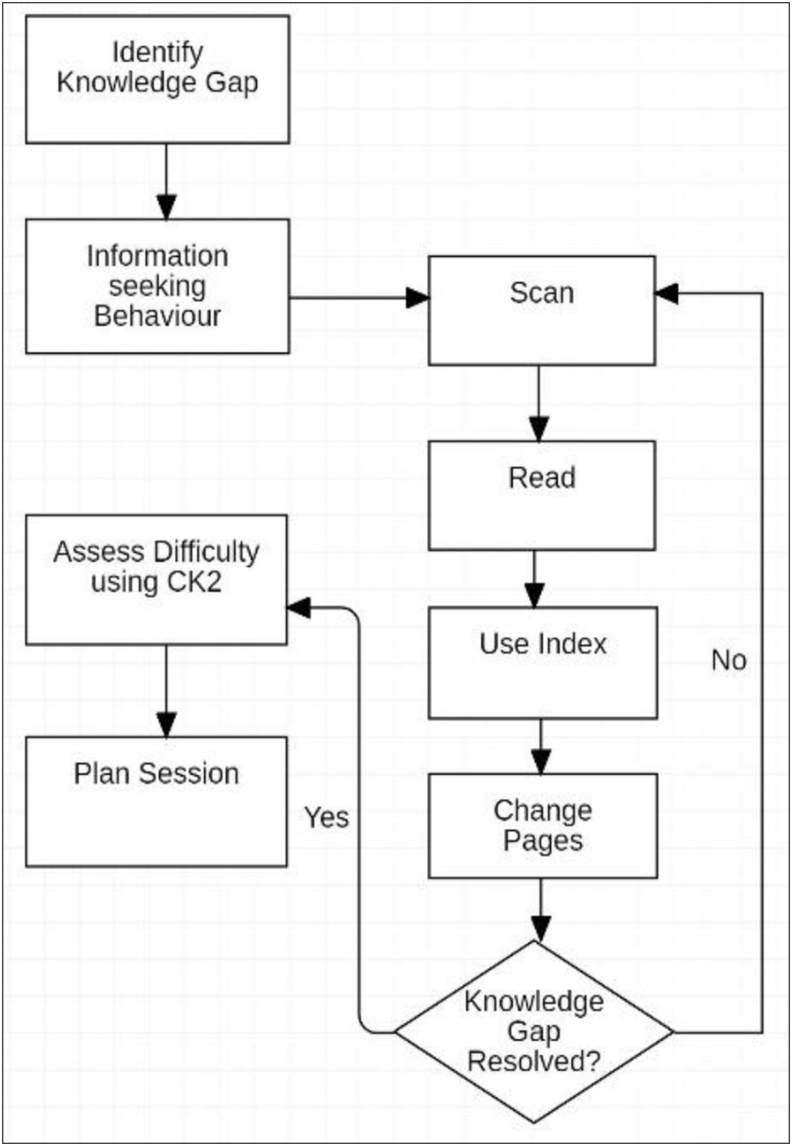
Pre-reading planning.

For the subsequent study sessions with the paper book, abbreviated planning was conducted at the commencement of each session (Figure 6). Within this context, 2 further patterns of planning behaviour were shown:

During pre-reading planning using the paper book, a brief evaluation of the text was conducted to set expectations for improvements to be made in the Knowledge State (KS) within this session. Content Knowledge (CK_2_) about the paper book/tool was again updated and used for evaluating the nature and difficulty of the material to be covered.

**Figure 7:**
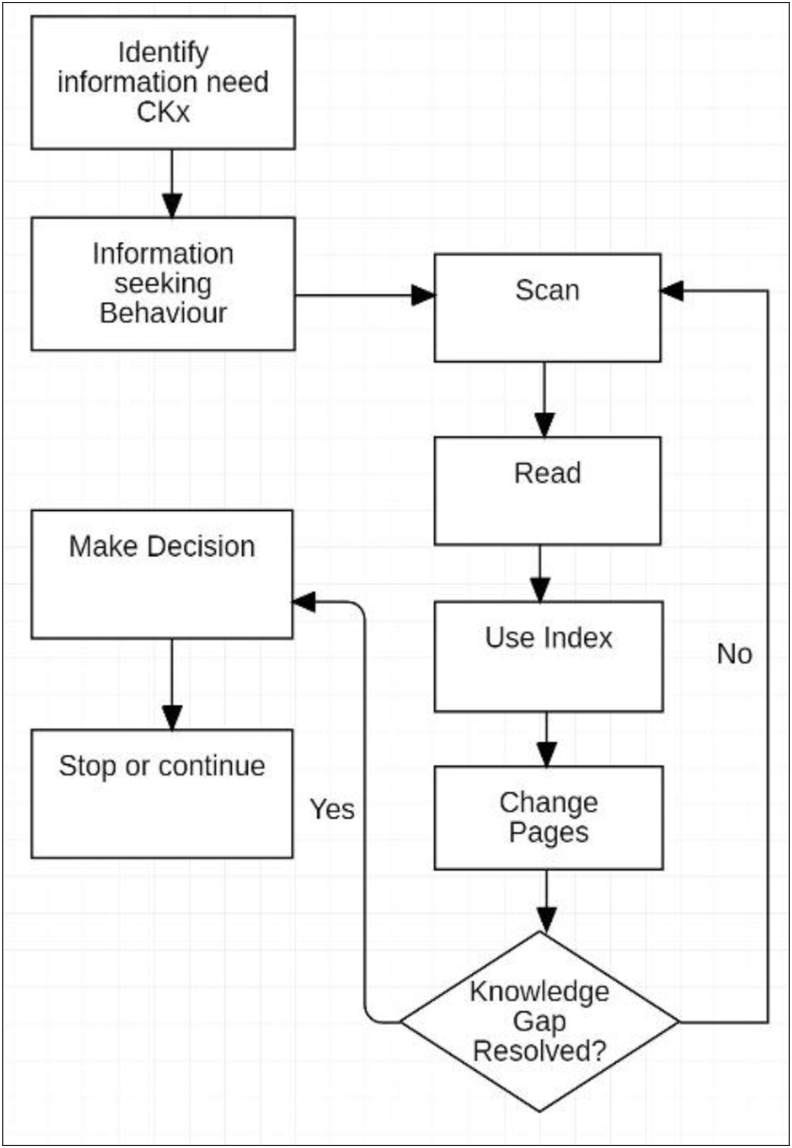
Decision process.

The decision process (Figure 7) was conducted during each session using the paper book at least once. The decision to continue reading was made based on expectations for progress during the session, fatigue in transforming the object, and by evaluating the content knowledge (CK) for the remaining work in the session. Interestingly, in each case the decision was made to continue to a logical stopping point using formatting in the book that separated concepts. In both workflows with the paper book, extensive use was made of operations including scanning, using the index and changing pages.

###### 3.1.4.3.4 Kobo

**Figure 8:**
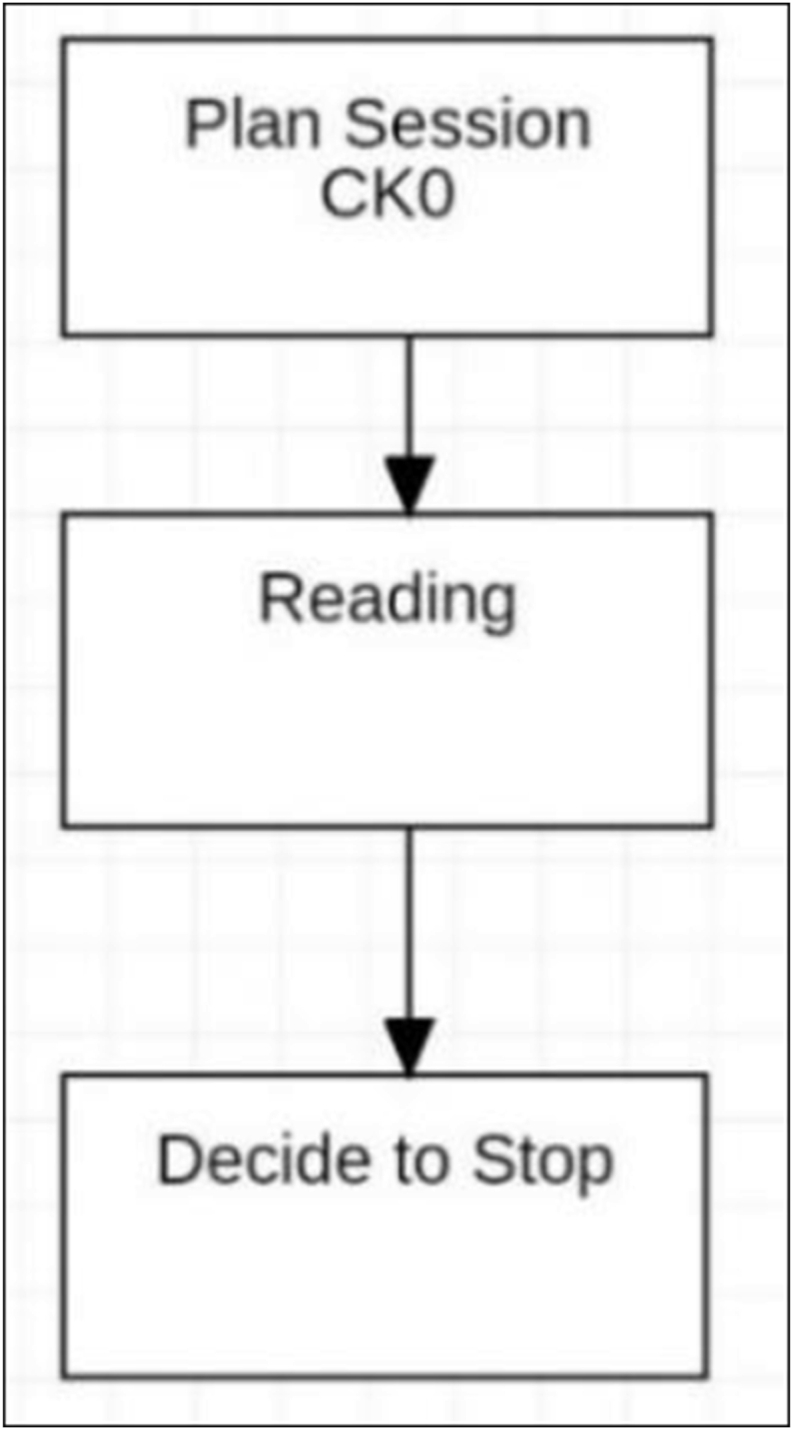
Kobo reading sessions.

Using the Kobo ereader, subsequent study sessions were again abbreviated (Figure 8) and workflow only resulted in forward linear reading. Initial attempts to determine progress or to update Content Knowledge were made, but these were unsuccessful due to the lack of supported operations and actions and were not repeated.

Without support for information seeking behaviour to obtain Content Knowledge, planning when using the Kobo consisted of noting the page number and the time. The decision to stop was made without being able to satisfy the information need in regard to the text content and was based on the time and page number only. In many cases this resulted in ceasing to read rather than the continuation observed when using the paper book. Within this simple structure of available actions and operations, session *planning* was not possible.

##### 3.1.4.4 Learning

Actions executed with the goal of *learning* also incorporated information seeking behaviour and decision making.

###### 3.1.4.4.1 Paper book

**Figure 9:**
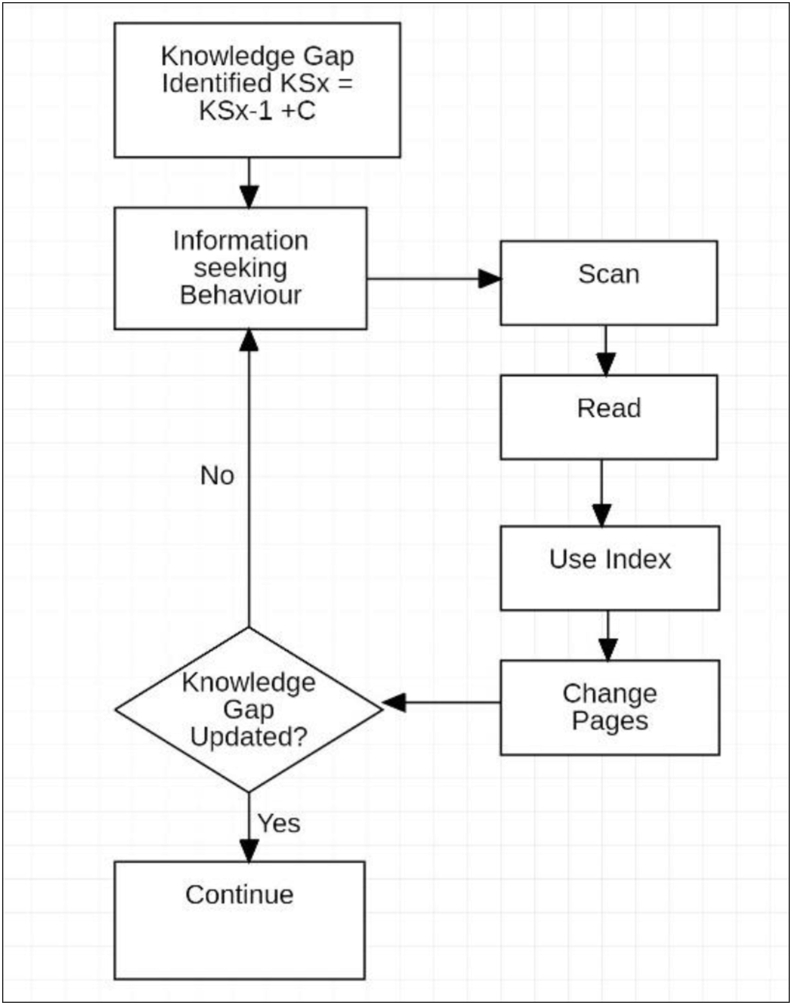
Learning flowchart.

Using the paper book, previously read material was frequently reviewed to confirm, consult or reconsider a concept. The flow chart for this workflow is shown below (Figure 9):

When using the paper book, the content in the text that was required by the information search was easily located and used to address the queries such as: *Have I learned this concept?* and *What was that point or reference?* Where it was considered worthwhile or productive in consideration of time and other priorities, the material was re-read using “focus” or “refer to colleague”. This sequence was also used for self-testing, and assessments of the updates to the Knowledge State against the established plan for progress within the study session were frequently made. Used in combination with *planning*, these actions facilitated self-regulation and metacognitive learning strategies as described by Brown et al. (1981) and Engestrom et al. (1999).

###### 3.1.4.4.2 Kobo

As noted in Stage 3, when using the Kobo, updates to the Content Knowledge were abandoned after the first study session due to the additional cognitive and time resources required to overcome the lack of supported operations needed for this process. In addition to this reduced Content Knowledge, the actions of scan, orient and contextualise could not be efficiently utilised to locate information in the text to confirm that content had been learnt. Review of concepts and updates to the Knowledge State was consequently determined to be too difficult, leading to this workflow also being abandoned after initial attempts. This insight into the weighing of priorities against time pressures, and the underlying related processes, adds descriptive reasoning to the work of Sidi et al. (2017) on the variation in the effect of time constraints on perceived importance. The decision to continue the study sessions without confirmation of an update to the Knowledge State or consulting previously read text was made in each instance. Self-regulation and planning were not possible using the ereader tool as it did not facilitate the processes for assessing study progress and learning in relation to the Ideal Knowledge State. Reading continued without support for metacognitive strategies and was perceived to be uninformed, frustrating and dissatisfactory. This experience supports the studies by Weisberg (2011) and Gregory (2008) which reported that students perceived reduced comprehension when using ereaders. When academic work was continued without assessment of the progress of learning, reading became notably faster which may have bearing on the research of Daniel and Woody (2013), in which student's achieved similar results with ereaders but required more time.

##### 3.1.4.5 Writing

**Figure 10:**
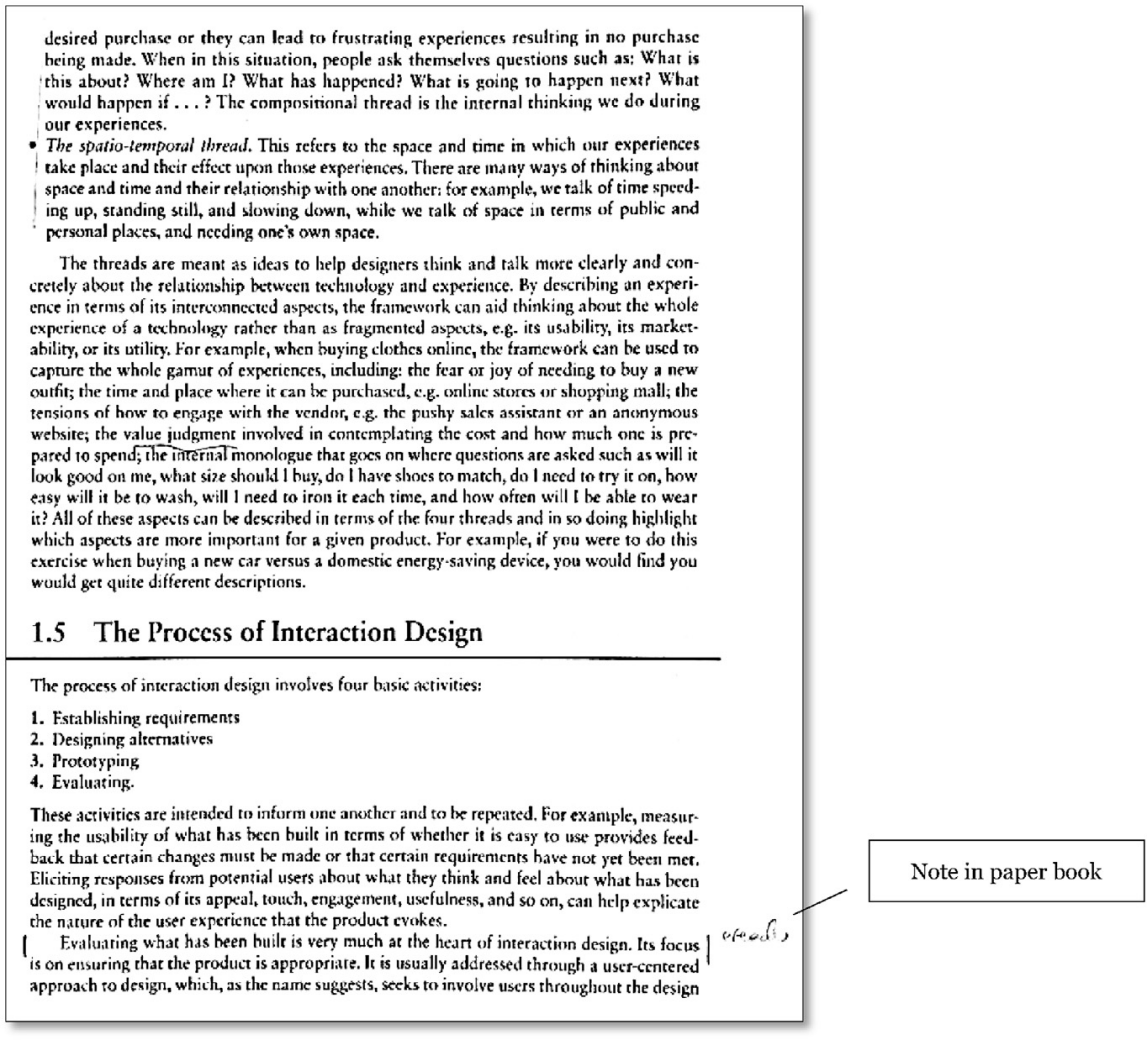
Annotations in paper book (Preece et al., 2015).

*Writing* was conducted as support for other action groups and to update the written Knowledge State. When using the paper book, writing within the academic work system consisted of making notations within the book (Figure 10), highlighting or making supplementary notes on a laptop or notebook. Notations made were complicated with variations in text, strength and the location of note for relevance with a wide variety of highlighting formats used to indicate the nature of the interest in the text (Figure 14). The Knowledge State, consisting of both the mental and written knowledge, was transformed through this and other specifically formatted annotations. In a study of student use of highlighting, Fowler and Barker (1974) found that annotation facilitates retention, and notes written through annotation also contribute to the production of further documentation that may form part of the ideal knowledge state.

**Figure 11:**
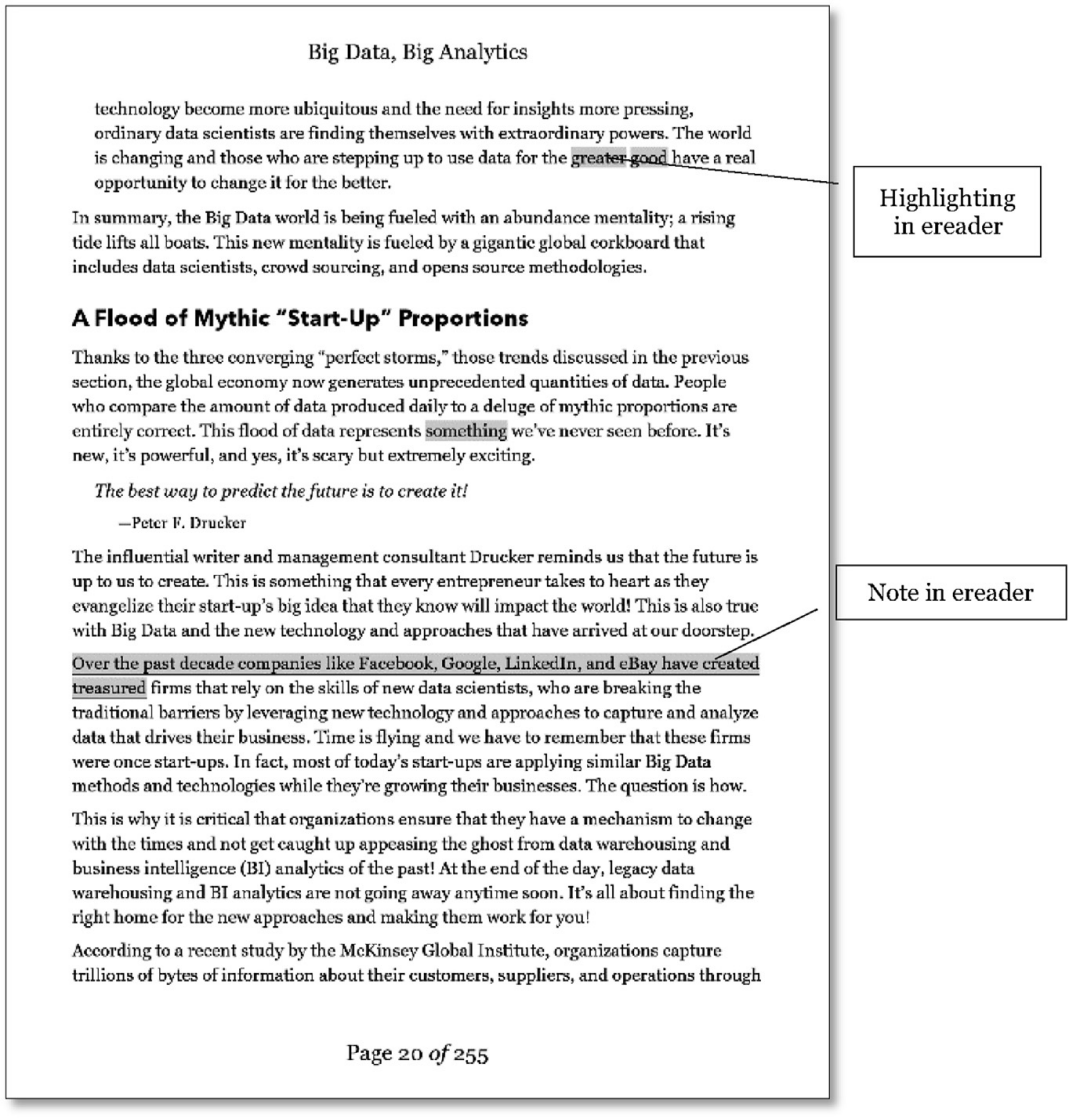
Annotations in Kobo ereader (Minelli et al., 2013). Copyright (2013) by John Wiley and Sons Ltd. Reprinted with permission.

Copyright (2015) by John Wiley and Sons Ltd. Reprinted with permission. Using the Kobo, the writing experience was restricted, and this was observed in the breakdowns occurring when implementing the writing operations (Stage 2). While text could be highlighted, variation in this was difficult to achieve, with a number of menu options required to apply highlighting styles. The Kobo ereader offers the ability to attach notes to the text which is an advantage over the paper book marginalia in clarity and volume that can be recorded. A problem was observed when attempting to find previously made annotations or use them to navigate within the text. The Kobo ereader added underlining to text to indicate the presence of a note (Figure 11) but the volume or nature of the annotations could not be easily reviewed or assessed. When information seeking behaviour was employed, navigation within the text or searching for a particular annotation was not able to make use of visible content and opening each note to evaluate for selection was prohibitive. The annotation option provided by the Kobo thus caused failures when used for information retrieval in all action groups as the visual prompt provided by varied paper book marginalia was not available. No further notes were made in the e-book using this method as they were perceived to be lost without sufficient prompting or recollection to ensure incorporation of this work product.

### 3.2 Analysis – journals and small documents

The investigation thus far has explored the effect of the change in media when reading a chapter of prescribed textbooks. To investigate the use of journals and other small documents within the social environment, the section stage of the experiment included all of the course materials for one of the enrolled courses. This material included lectures, worksheets, journals and other documents used for one of the course modules in the following numbers:

• Lectures: 26
• Journals: 2
• Worksheets: 3
• Other Pdf documents: 1
• **Total documents 32**

#### 3.2.1 Stage 2 – identifying operations and conditions

Original document formatting was retained in the importing process as the documents were not converted to ePub or other proprietary formats. No font changes, annotation or other ereader functionality was available for the unconverted documents and hardware ereaders do not display colour. The large number of documents meant that searching processes were required to locate a required document from within the list. “Identify Document” was employed with the previously identified operations in varied sequences and goals when using smaller documents.

Breakdowns were again frequently observed in most operations using the ereader and effects varied from the experience of the e-book (Table 4). The slow scrolling of the ereader had less impact as the documents were smaller and information searching was more localised. In the case of reading lectures, the documents were more sparsely populated, with more variable formatting that was preserved in the imported document. Information was condensed by many bullet points and subheadings and navigating to locate points within a document was more easily facilitated by these points and their spatial and conceptual relationship to each other. Journals and other documents offered similar benefits with smaller document size and retained formatting, which reduced scrolling requirements and facilitated information searching.

**Table 4:** Identifying journal operations.

Operations	Kobo
Read text	Yes
Scan Page	Yes
Scroll/turn page	Yes
Identify document	No
Underline text	No
Highlight text	No
Write in book	No

**Figure 12:**
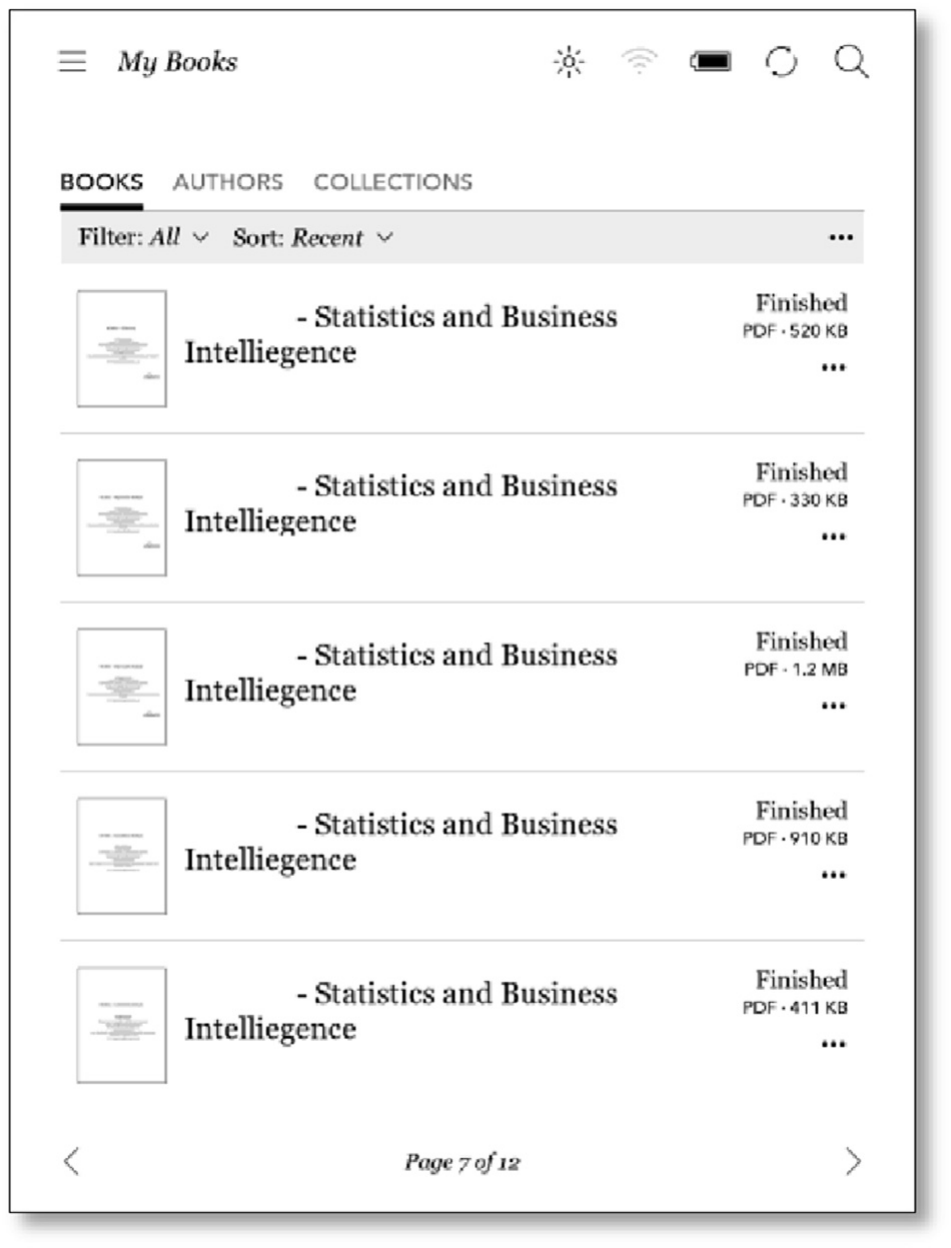
Kobo screen showing imported lectures.

Information retrieval was experienced very differently when trying to locate information within an unknown lecture. Most identifying information for imported documents was condensed during the import process, and it was not possible to separate the content of one lecture from another. Figure 12 shows a Kobo screen of the imported lectures, demonstrating the lack of discriminating information remaining for each lecture after the import process (the course code reference has been removed but it is also identical). Many lectures (between five and ten) needed to be opened each time in order to locate required information when searching. In many cases, navigation was assisted by the size of the document in KB and a recollection of the content associated with that size. Date ordering was not helpful as it referred to the import date, and the documents could not be renamed. An attempt at organisation was made but this was only possible to one level of sub-folders and the amount of work required to sort these documents resulted in a minimal improvement that was determined to be unproductive. Journal articles were easier to locate as the naming was more variable, however this did not provide sufficient information to prevent many re-openings and scanning.

While working with these documents, notes were made on an accompanying laptop and the connection between the notes and the studied material was considered less than that provided by spatially located marginalia. At the date of writing no ereaders or ereader applications support annotation for imported documents.

#### 3.2.2 Stage 3 – The ideal object

While evaluation of the suitability of the prescribed e-book text was not needed, the use of the smaller articles was more subjective, and their direct relevance for use in obtaining the ideal knowledge state was often evaluated. This requirement was observed in an additional series of processes as outlined:

(6.1) $$\begin{array}{ccccc} & & {\text{CK}}_{\text{0}} & & \\ & & ↓ & & \\ {\text{KS}}_{1} & ↔ & {\text{CK}}_{1} & ↔ & {\text{IKS}}_{1} \end{array}$$

For each document, the Content Knowledge (CK_0_) was updated by locating the document, opening, scanning and reviewing the content making strong use of available formatting. The updated Content Knowledge (CK_1_) was then used to assess the document for relevance and usability in transforming the current Knowledge State (KS). An example of this included opening a word document for “Questions asked Week 1”. This was easily scanned, and it was observed that it provided information on accessing the distance services. While this was determined not to be of direct use for the IKS, the content was noted for assistance at a later time. A journal article supplied in Week 5 was opened and the heading, abstract portions of the text were scanned and reviewed to enable evaluation of the content. The journal article was determined to be a helpful overview but the priority for close reading in relation to the IKS was low. The decision was made within the social environment to return to a close study of this article if time permitted. The limited display information on the ereader screen (Figure 16), and the lack of customisation options available for imported documents in the ereader, led to significant extra work, and repeated applications of “Identify Document” in assessing of the content of each document.

#### 3.2.3 Stage 4 - Organisation

Analysis of the operations and actions used within the journal experiment revealed a new goal when working with multiple, varied format documents (Table 5).

**Table 5:** Organisation action group.

Group	Action	Description	Kobo
Organisation	Information seeking	Review or scan content for relevance to knowledge gap or ability to cover in reading session. Review progress.	No
	Deciding relevance	Use knowledge of content.	No

To organise and utilise the smaller written articles, actions and operations were completed to scan and review the articles and to select for further reading. These steps contributed to the goal of organisation, which was utilised to facilitate planning and learning with the many documents to achieve the Ideal Knowledge State. Two new workflows were observed and are presented below.

**Figure 13:**
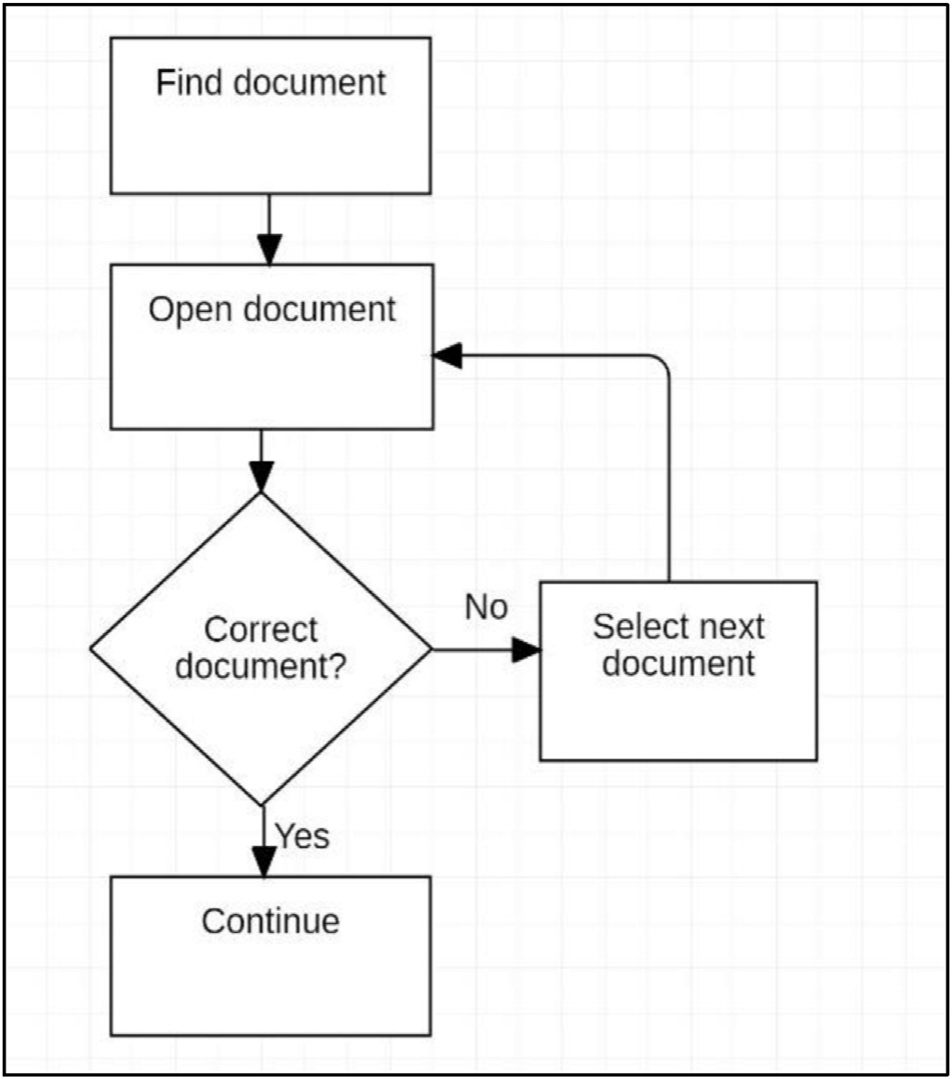
Find document.

**Figure 14:**
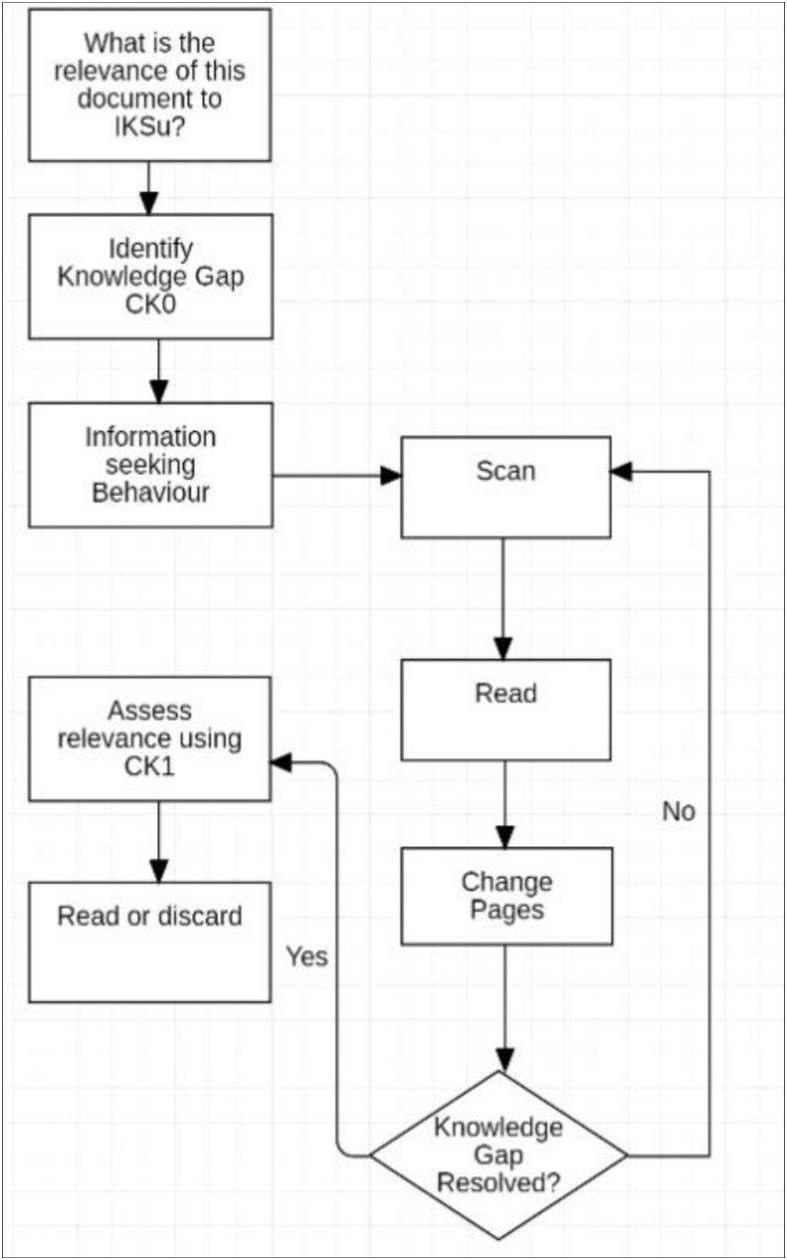
Decide relevance.

Breakdowns occurred in all of the actions within the Organisation group which effected the workflows of “Find Document” and “Decide relevance” (Figures 13 and 14). As the documents could not be effectively curated or labelled, locating particular documents required opening and reviewing content many times, resulting in iterative applications of information seeking behaviour. This work needed to be repeated in each study session for *planning* sessions and for more complex *learning* processes. Once the desired article was located, the decision making and planning was conducted using scanning, reading and other actions. This behaviour was noted by Guthrie (2012): “Journals are generally not read in their entirety but articles of interest are selected, which are relatively short and available in a common PDF format and lend themselves to printing if required”. The work of selection, assessment and categorisation could also not be retained within the ereader format, decreasing the efficiency of this system.

## 4 Discussion

### 4.1 Comparison study

**Figure 15:**
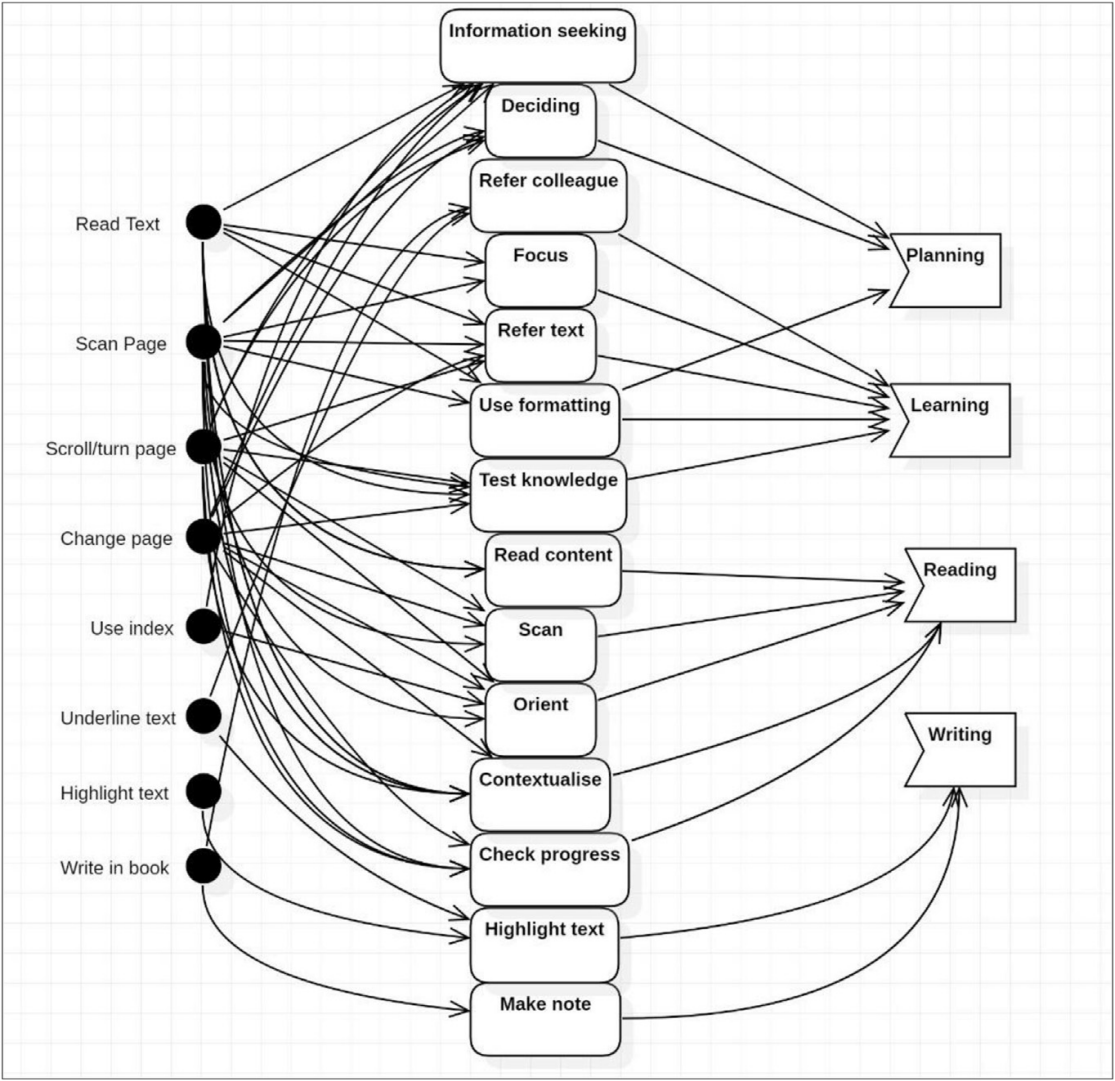
Skills mapping with Paper Book.

**Figure 16:**
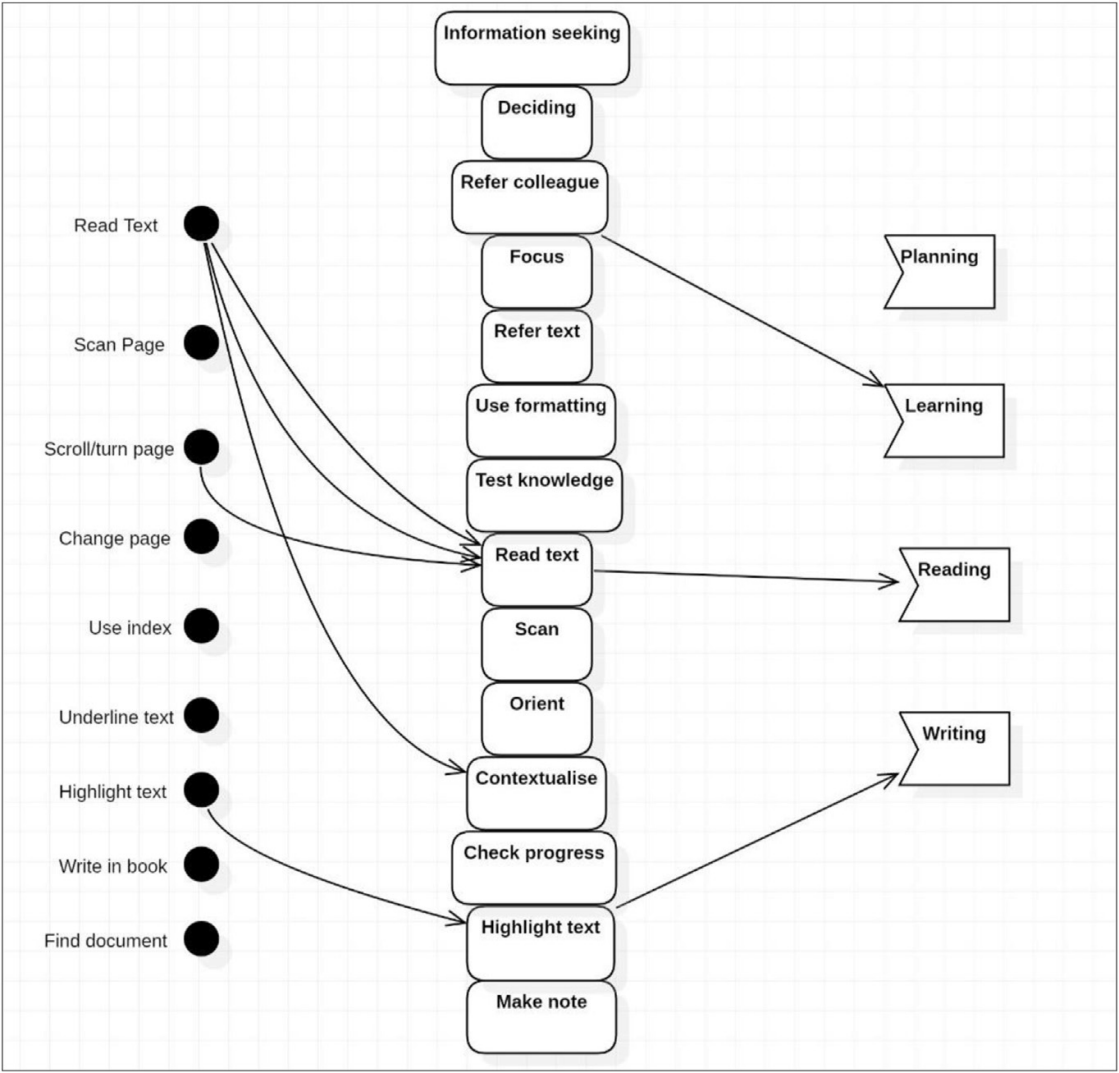
Skills mapping with the Kobo ereader.

The comparative analysis of the mapped activity using the ereader clearly showed the adaptations required when using the Kobo ereader as the tool for mediating this activity. Figure 15 shows the complexity of operations and actions that were demonstrated during the experiment when using the paper book and Figure 16 outlines the processes utilised within the experiment when using the Kobo ereader. The impact of the breakdowns in operations can be seen in the significantly reduced complexity of processes utilised as well as in the effected workflows mapped in the previous analysis.

The reduction in the complexity of processes and workflows observed when using the ereader is severe and equates to a reduced ability to apply the metacognitive processes and skills required for learning, reading (Brown et al., 1981; Engestrom et al., 1999) and *academic work*. In Engstrom's theory on the successful expansion of an activity (1999), the incorporation of new technology should disrupt and improve the existing processes. In this experiment, the effect of the change in the tool can be seen to significantly reduce processes without any substantial improvement. Engstrom's approach is utilised in studies in Activity Theory investigations into the implementation of technology into the building industry (Lu et al., 2018) and the incorporation of a Virtual Learning Environment by Dublin City University (Blin and Munro, 2008). In both cases Activity Theory was applied to explore the use of the deployed technology and to identify the experiences of staff in the use of the new and traditional processes. In each case, the negative impact of the new technology on existing processes was revealed, and this was found to be causing resistance to adoption. In this experiment, the impact of the reduction in processes is seen in the student's perceived lower outcome or reduced efficiency, which would result in resistance to the adoption of e-books.

### 4.2 Journals and small documents

In the findings from literature studies (Hobbs and Klare, 2015; Liverpool University, 2010; Swain, 2010; Tracey, 2018; Zhang et al., 2017), students exhibited different behaviour with shorter text and this was upheld within this investigation. The critical point of failure for reading journal articles on the Kobo resulted from an inability to organise documents and to conduct planning without an unacceptable level of inefficiency within the social context. The variation in the causal factors for the resistance to e-books shown by the same student when using shorter documents and full-length e-books, explains the difficulty in establishing conclusions about the resistance to e-books without a detailed understanding of the behaviour.

### 4.3 Relation to existing findings

The comprehensive mapping of processes utilised by the student in this experiment showed the individuality of study practices (Brown et al., 1981; Gier et al., 2011) and that the response to the change in media was particular to these practices. In this investigation, the student perceived that work was “lost” or the opportunity to record thinking was reduced, leading to a comparatively higher cognitive load when information needed to be retained in working memory (Souza et al., 2018). Background theory notes the importance of annotation for learning from text (Fowler and Barker, 1974) and in this instance the reduced capability was perceived to lead to reduced volume and quantity of learning and other academic work product. Given the highly individualised nature of annotation styles, it is expected that the effect of this reduction in capability may vary significantly in response to individualised annotation practices, contributing to inconclusive literature findings in related to this e-book factor.

Navigation was engaged extensively when using the paper book for the action groups of *planning*, *learning* and *reading*, confirming the findings of background studies that note the importance of this process for learning with documents (Lovelace and Southall, 1983; Mangen et al., 2013; Rothkopf, 1971). When using the ereader, breakdowns in operations that required navigation such as scan and change page, produced critical failures, and the significant variation this created in workflows was shown in the comparison experiment (Section 3.1). Background studies recognise that students' reading strategies vary widely and navigation skills are complex (Dillon, 1994; Gier et al., 2011) and the effect of breakdowns in operations that support navigation is dependent on this variation. This finding offers an explanation for the range of responses to navigation problems reported in literature studies.

Comprehension using the e-book was perceived by the student to be reduced when breakdowns in operations prevented the implementation of actions within the *learning* group for self-testing and accessing previous topics. This experience corresponded to student reports of perceived lower comprehension and resulted in the student's perception of faster, shallower reading. A perception of reduced comprehension was not observed when testing with the smaller documents and the breakdowns in information retrieval experienced in this experimental section led to a perception of reduced efficiency. The variation in perception observed during the investigation correlates with the range of research findings in relation to the perception of comprehension by students using ereaders and digital text documents.

This mapping of processes revealed the effect of the social context in the student's allocation of time resources to resolving issues with the ereader. Information needs were unable to be readily resolved and critical points occurred at which the student decided to abandon the process as described by Tikhomirov (1999). Students are time poor in a competitive environment (Fyfe, 2014; Gibson and Gibb, 2011) and the outcome was perceived by the student to be superior and more efficient when using the paper book than the ereader in this context. The analysis provided reasoning for the resistance to adoption of e-books by demonstrating the basis for the student's perceived lower outcome when utilising the restricted learning strategies against the time constraints of the social environment. Delgado et al. (2018) found that the imposition of a time restriction led to greater disadvantage in comprehension when learning with digital text and this investigation offers potential causality for this, and other studies on this effect (Lenhard et al., 2017; Sidi et al., 2017; Stole and Anne Mangen, 2020). This experiment showed the importance of the social context on investigations into e-book use by students.

### 4.4 Overall

**Figure 17:**
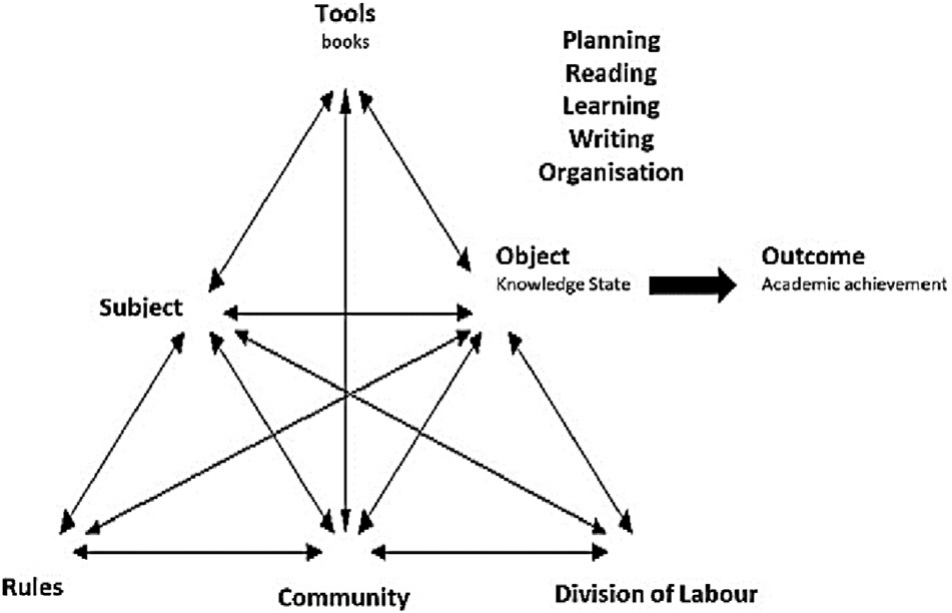
Activity Theory model of Academic Work. Adapted under CC-by-SA-3.0 (Bury, 2012).

The activity was divided into five key action groups: *planning*, *reading*, *learning*, *writing* and *organisation*, all of which occurred in combination, and iteratively, to transform the cognitive and written knowledge state into the perceived ideal state (Figure 17).

Analysis with activity theory reveals the range of actions and workflows that were conducted when using text sources for academic work, and the importance of the metacognitive requirements and strategies utilised for learning through reading (Brown et al., 1981; Hattie, 2009). The wide variation in effect and impact caused by the use of the ereader and the digital document type suggest that addressing the problem of low adoption rates through changing content delivery method or correction of specific technological factors will be challenging. The Activity Theory mapping of the observed academic processes was extremely complex and required support for action groups other than linear reading. In the investigations by Lu et al. (2018) and Blin and Munro (2008) into the adoption of technology, Activity Theory analysis revealed the lack of adequate support for the new processes required for the disrupted activity systems. The findings from these studies recommended the implementation of distributed support practices to ensure the successful evolution of enhanced work systems. In the case of e-books, improved support for the technologically enhanced activity should include improvement of the functional support within the application. As discussed, student processes are highly individualised and the response to corrective changes to the existing technology are likely to be similarly difficult to predict.

The comparative impact of the ereader observed in this experiment was severe and placing this example within literature suggests that this is an indicative example of the disrupted academic experience with this technology. Sales of academic e-books have plateaued and extending the results of this analysis to the wider use of e-books, it is proposed that the reason for this is that e-book reader technology is not well designed to support academic study and learning. In an AT investigation by Sun (2016), the resistance to information technology was investigated using a framework that identified the effect of tool experiences, task situations and user characteristics on the user's choice of tool. In this research, Sun found that students were reluctant to adopt a technical tool when they experienced a low sense of control or cognitive harmony which is supported by the findings of this investigation. When investigating the adoption of technical products into environments that require a high level of cognitive work, the impact of insufficient support for the processes is frequently found to contribute to user resistance (Bodker, 1996; Rundo et al., 2020; Zheng et al., 2005). To address this issue, Sun (2016) recommended redesign and better support of technical innovations and it is proposed that innovative product design or re-engineering of e-book technology to address the requirements of the academic work domain may result in a far superior fit as a student-centric product. In a study on the approach to innovation, Smith (2011) state that new products are only introduced when there are “major conflicts” between users and existing products, and this investigation finds this to be the case. A successful ereader product would need to better support academic study processes and provide advantages that a paper book cannot facilitate to improve the perceived usefulness ratio that is required for the successful adoption of technology (Venkatesh and Davis, 2000). Where it is not possible to replace functionality provided by the physicality of a paper book, the resistance/reward ratio for the acceptance of a new product could be addressed through innovative exploitation of technology to offer alternative enhancements or functionality to the academic. While supporting a different media format, comparisons can be made with the adoption of the iPod which revolutionised the music industry (Pattuelli and Rabina, 2010). The iPod provided music listeners with a new way to enjoy music through redesign of the listening technology and users quickly realised provided rewards that expanded their listening experience. Subsequent iPod models developed iteratively in response to evolving public usage, and market penetration for digital music has reached 75% (IFPI, 2019).

A means of reading via advanced technology has been desired and proposed by academics for more than 70 years (Brown, 1930; Bush, 1945). In 1963, Engelbart (1963) described in detail the potential that may be possible should technology succeed in effectively addressing the work requirements of academics. These writers described a portable device that would facilitate and assist their work with the rapidly growing range of writings and research publications. Redesigning ereader and e-book technology through analysis of the academic workspace and synthesis of a new innovative technological product solution offers an alternative pathway to address the low adoption rates of e-books. Options could include:

• A platform independent application – this would make good use of the increasing availability of documents and e-books. Formatting would be retained and scroll speed issues resolved.
• User-centric design – most ereader applications are structured for the delivery of retail services or university content. A student focus would incorporate organisational functionality for imported documents and student work. Design should focus on identifying and supporting student cognitive requirements.
• Enhanced annotation capability including Optical Character Recognition – fully customisable annotation with links to existing work, imported documents and internet resources. Links could be maintained for later research, bibliographies and extended searching.
• AI enhancements for individualised searching and concept analysis.

A detailed work analysis and iterative user-focused design could provide significant advantages for students and academics. An innovative product that resolves conflicts where observed and provides enhancements offers a potential pathway to improving the adoption of e-books.

## 5 Conclusion

The use of AT/AE methodology provided results that address the “problem of e-books” as follows:

1. Why are the adoption rates for student use of e-books and ereaders so low?

The experiment of using an e-book on an ereader revealed a significant negative impact on complex individualised processes when compared to a paper book. This impact is sensitive to the social environment and student and is specific to the use case of academic study.

2. What are the effects of using digital text or ereaders for learning and academic study, and how do they contribute to the low adoption rate?

The use of the digital documents on an ereader led to a perception of reduced quality and efficiency in learning and academic work due to reduced processes and workflows. The breakdowns experienced are representative of findings from literature studies and are likely causal factors for the low adoption rates of e-books.

3. What changes can be made to e-book and digital text delivery to improve the adoption rates?

The processes used within the academic domain were poorly supported and redesign or re-engineering of a new ereader or e-book product that is specifically designed for academic work requirements has merit as a pathway to improve low adoption rates.

### 5.1 Limitations

Diagnosis of the problem with ereaders was performed using results from an autoethnography investigation by a single student. The result is specific for particular environmental conditions and this must be considered for any application of findings. While the results of the analysis and application of theory extend the perspective of the findings, further investigations to explore this issue should be conducted, and analysis of the use of ereaders and e-books by undergraduate, internal and exam-based student groups would provide insight into varied priorities, learning strategies and resultant practices. Incorporating professionals that utilise academic practices within their workplaces would allow further observation of the effect of varied social communities. The analysis is affected by the selection of the technology, and further development of these findings should consider the improvements available in ereader and e-book technology.

## Declarations

### Author contribution statement

K. Kirby: Conceived and designed the experiments; Performed the experiments; Analyzed and interpreted the data; Contributed reagents, materials, analysis tools or data; Wrote the paper.

M. N. Anwar: Contributed reagents, materials, analysis tools or data; Wrote the paper.

### Funding statement

This research did not receive any specific grant from funding agencies in the public, commercial, or not-for-profit sectors.

### Competing interest statement

The authors declare no conflict of interest.

### Additional information

No additional information is available for this paper.

## Footnotes

1 – IKS_u_ is initially vaguely defined. Clarity in this object is also increased through iterative information seeking sessions with CK, comparing progress and updating the KS. The value of the IKS_u_ in achieving the Outcome_i_ is the critical factor for decision making, as is the relationship between IKS_p_ and IKS_e_. Further development in this area is outside the scope of this work.

2 An analysis of the relationship between Content Knowledge and Content for the purposes of addressing a Knowledge Gap is beyond the scope of this works.

3 Where KS_0_ is considered a subset of KS_1_ per Knowledge Space theory (Doignon and Falmagne, 1985).
